# Concave sparsity-assisted time-reassigned multisynchrosqueezing transform for early-stage weak fault feature enhancement with applications to bearing fault diagnosis

**DOI:** 10.1371/journal.pone.0360733

**Published:** 2026-10-16

**Authors:** Delong Zhang, Yubo Ma, Hongan Wu

**Affiliations:** 1 Hubei Key Laboratory of Power System Design and Test for Electrical Vehicle, Hubei University of Arts and Science, Xiangyang, P.R. China; 2 Hubei Longzhong Laboratory, Hubei University of Arts and Science, Xiangyang, P.R. China; 3 Key Laboratory of Metallurgical Equipment and Control Technology, Ministry of Education, Wuhan University of Science and Technology, Wuhan, China; 4 Hubei Key Laboratory of Mechanical Transmission and Manufacturing Engineering, Wuhan University of Science and Technology, Wuhan, China; Southern University of Science and Technology, CHINA

## Abstract

Early-stage defects in wind turbine bearings often generate weak impulsive signatures that are easily obscured by strong background noise, posing a significant challenge to accurate extraction of fault-related features. To overcome this limitation, a novel concave sparsity-assisted time-reassigned multisynchrosqueezing transform (CSA-TMSST) is developed to improve the extraction of weak fault-related signatures. First, a non-convex sparse optimization model incorporating the generalized minimax-concave (GMC) penalty is established to reconstruct fault impulsive components from the raw signal. The introduced GMC-based sparse regularization enhances signal sparsity, enabling the accurate extraction of impulsive fault signatures. Subsequently, TMSST is employed to characterize the reconstructed signal in the time-frequency (TF) domain, yielding a more concentrated time-frequency representation (TFR). Furthermore, the iterative reassignment mechanism is utilized to enhance the concentration of TF energy distribution, thereby yielding a clearer TFR of transient fault features. Finally, characteristic fault frequencies are identified via spectral analysis. The results demonstrate that CSA-TMSST is capable of extracting weak fault-related signatures and producing high-quality TFRs under strong noise conditions, confirming its effectiveness and superiority.

## 1 Introduction

Wind turbine bearings are critical components of the drive system, and their early faults are often one of the major causes of equipment shutdown. Slight defects in rolling bearings at the early stage may rapidly evolve into severe faults within a short period, leading to equipment shutdown, reduced wind power generation efficiency, and significant economic losses. Studies have shown that when localized defects appear on the bearing surface, periodic transient impulsive responses are generated as the rolling elements traverse the defective region. These impulsive signals usually contain critical fault-related information [1,2]. However, under actual operating conditions, the impulsive energy generated by early faults is often extremely weak and easily masked by strong background noise. Moreover, vibration signals are often affected by coupled vibrations from other rotating components as well as electromagnetic interference, making weak fault characteristics more difficult to identify. Therefore, it is highly important to develop effective diagnostic methods for early weak fault detection to enhance the operational stability and reliability of equipment [3–5].

The rapid progress of time-frequency analysis (TFA) has provided new research directions for transient impulsive fault diagnosis. By simultaneously characterizing signal features in the time-frequency (TF) domain, this approach enables effective analysis of transient signals [6,7]. In recent years, TFA techniques based on signal decomposition and time-frequency representation (TFR) have been extensively utilized for bearing fault diagnosis. Within decomposition-based approaches, Selami et al. [8] applied empirical mode decomposition (EMD) to break down vibration signals into multiple intrinsic mode functions (IMFs), thereby capturing fault-related components and facilitating effective identification of bearing faults. Zheng et al. [9] proposed the uniform phase EMD (UPEMD) approach, which mitigates noise interference by introducing uniformly phased sinusoidal signals and successfully identifies bearing faults. However, EMD-based signal decomposition methods are prone to mode mixing, which makes it difficult to effectively separate weak transient impulsive components. To mitigate this limitation, Dragomiretskiy et al. [10] proposed variational mode decomposition (VMD) by constructing a variational optimization framework, which mitigates the mode mixing problem to some extent. Furthermore, Ni et al. [11] established a fault information-guided VMD (FIVMD) approach by introducing a generalized Gaussian cyclostationary (GGCS) model together with a generalized Gaussian stationary (GGS) representation into the VMD framework, thereby enhancing its ability to extract weak fault impulsive signals. Nevertheless, VMD and its improved variants generally rely on parameter settings and iterative optimization procedures, and may still suffer from insufficient decomposition stability under strong noise interference conditions.

At present, another important research strategy is to directly characterize signal energy patterns in the TF plane, as impulse-like components in this TF domain are closely associated with fault impacts. Based on this concept, Chen et al. [12] constructed a generalized dispersive model (GDM) to progressively extract fault-related transient impulsive components, and further developed a generalized dispersive mode decomposition (GDMD) framework on this basis, thereby obtaining high-quality TFRs. Zhou et al. [13] leveraged the excellent TFR capability of the transient-extracting transform (TET) to preprocess raw signals, thereby enhancing the concentration of transient impulsive energy in the TF plane and achieving bearing fault diagnosis. Subsequently, Yu et al. [14] further developed the second-order TET (STET), which utilizes second-order phase information to achieve a more accurate TFR of nonstationary signals, effectively improving the TF concentration of transient components. In addition, Bao et al. [15] developed the generalized TET (GTET) by establishing a generalized transient-extracting framework, which further enhances the TF concentration capability of fault impulsive features under strong noise environments, enabling more effective processing of impulse-like signals. Although the aforementioned methods can improve the representation capability of TF features, strong noise interference often causes the TF energy of early weak faults to become dispersed, resulting in reduced TF resolution and hindering the accurate characterization of fault-related information. Consequently, researchers have developed the synchrosqueezing transform (SST) and its enhanced versions to address this issue. For example, Daubechies et al. [16] proposed SST, which effectively improves the concentration of TF energy by reallocating wavelet coefficients. Subsequently, Yu et al. [17] developed the multisynchrosqueezing transform (MSST) based on SST. This method further improves the TF resolution through iterative multiple SST operations applied to the TFR. However, the issue of insufficient TF energy concentration still exists at certain specific frequency points. To address this issue, Yu [18] further proposed an improved MSST (IMSST) method, in which the instantaneous frequency estimation is refined via two iterative rounding procedures, thereby substantially alleviating the problem of weak energy concentration in MSST. Nevertheless, this method still exhibits certain limitations in extracting weak fault features. Furthermore, He et al. [19] developed a time-reassigned SST (TSST), in which the TF spectral energy is compressed along the time direction and reallocated it near the true group delay (GD), thereby obtaining a clearer TFR. However, when processing impulse-like signals with rapidly varying frequencies and large amplitudes, the energy of the extracted impulsive components becomes severely dispersed. To further improve the concentration of signal TF energy and accurately capture impulsive components in signals, Yu et al. [20] introduced the time-reassigned MSST (TMSST), which enhances TF concentration by iteratively reassigning energy while incorporating the time-varying nature of the instantaneous frequency. However, in real-world scenarios, vibration signals usually contain substantial noise and irrelevant interference components, while noise is generally diffusely distributed over the TF plane. Such interference can easily mask the weak impulsive features generated by incipient faults, significantly degrading the TF characterization capability of methods such as TMSST and making early fault diagnosis considerably more challenging. Therefore, prior to TFA, effective noise suppression and feature enhancement of the raw signal play a crucial role in enhancing the accuracy of fault feature extraction [21].

Recently, preprocessing approaches employing sparse representation have attracted considerable attention and been applied to fault feature enhancement [22,23]. In early bearing fault diagnosis, fault impulsive signals typically exhibit strong impulsiveness and periodicity, and thus possess pronounced sparse characteristics [24,25]. In contrast, background noise is mostly randomly distributed and generally does not exhibit sparse structural properties. Based on this difference, many researchers have constructed sparse optimization models to separate fault-related impulsive components from noisy signals. For instance, Zhang et al. [26] developed a bearing fault diagnosis approach using generalized logarithm (G-log) regularization. By introducing a generalized logarithmic non-convex penalty function, the sparsity of the signal representation is reinforced, thereby effectively enhancing the reconstruction performance of fault impulsive components as well as the noise suppression capability. Zhang [27] developed a bearing fault feature extraction approach that integrates the flexible analytic wavelet transform (FAWT) with non-convex sparse regularization. This method leverages the excellent TF coverage capability of FAWT to match fault impulsive components, thereby effectively improving early fault diagnosis accuracy. In addition, based on Moreau envelope sparse optimization, Qiu et al. [28] proposed a transient impulsive component extraction method. This method imposes non-convex sparsity constraints on both the matrix elements and the singular value vectors in the TF domain, thereby effectively alleviating the amplitude underestimation problem inherent in conventional sparse models. Consequently, it further improves the reliability and effectiveness of extracting fault-related information. More generally, weak fault feature extraction can be regarded as a process of structuring a complex high-dimensional signal representation space, such that diagnostically relevant feature structures can be effectively distinguished from noise and irrelevant interference. For complex high-dimensional problems, decomposing the original representation space according to specific structural characteristics provides an effective means of reducing problem complexity and enhancing target distinguishability. For example, Lan et al. [29] decomposed a multiclass classification problem into multiple relatively simple binary classification subproblems using a class-binarization strategy, achieving favorable performance in terms of classification accuracy and robustness. The underlying principle of structurally decomposing complex problems into more tractable subproblems provides useful theoretical insight for the hierarchical representation and progressive enhancement of weak fault features. For bearing vibration signals under strong noise conditions, fault-related impulsive components can first be highlighted from the high-dimensional mixed signal by exploiting their sparse structural characteristics, followed by refined TF characterization of the enhanced signal, thereby progressively improving the distinguishability between weak fault features and background interference.

Inspired by the aforementioned studies, a concave sparsity-assisted time-reassigned multisynchrosqueezing transform (CSA-TMSST) approach is developed to enable early-stage fault feature extraction in rolling bearings. The proposed framework integrates a non-convex sparse enhancement strategy based on the generalized minimax-concave (GMC) penalty with the iterative time-reassignment mechanism of TMSST, thereby exploiting their complementary advantages in weak fault impulse enhancement and high-resolution TFR. The main contributions of this work are summarized as follows:

(1) A GMC-based non-convex sparse enhancement strategy is introduced to reconstruct weak fault-related impulsive components under strong noise. By exploiting the sparse structural characteristics of early-stage bearing fault impulses, the GMC penalty is incorporated into the sparse optimization framework. This strategy suppresses background noise while effectively alleviating the amplitude underestimation caused by conventional convex sparse regularization, thereby enhancing weak transient impulsive components.
(2) A synergistic CSA-TMSST framework is constructed to integrate sparse enhancement of weak fault features with high-quality TFR. Fault-related sparse impulsive structures are first enhanced through GMC-based non-convex sparse reconstruction, after which the iterative time-reassignment mechanism of TMSST is employed to further concentrate the TF energy of transient impulses. In this manner, noise suppression, impulse preservation, and highly concentrated TFR are jointly achieved, thereby improving the extraction and characterization of weak fault features under strong noise conditions.
(3) The effectiveness of CSA-TMSST is validated using simulated signals and two experimental bearing datasets, with comparisons against EMD, VMD, TMSST, and the L1-norm-based TMSST (L1-TMSST). The results demonstrate that CSA-TMSST produces clearer and more highly concentrated TFRs and more effectively extracts weak fault-related impulses and their associated features under strong background noise.

The remainder of this paper is organized as follows. Section 2 introduces the theoretical foundations of TMSST and GMC, and details the implementation of the CSA-TMSST. Section 3 evaluates the performance of CSA-TMSST through simulation analyses. Section 4 verifies its effectiveness through two experimental cases. Section 5 concludes the paper.

## 2 Theoretical foundations

### 2.1 Theory of TMSST

To understand the principle of TMSST, this paper begins with its research object and constructs a signal model with time-varying components, which is expressed as follows:


$$\begin{array}{c} \tilde{s}\left(\omega \right)=A\left(\omega \right)\cdot e^{i\cdot \theta \left(\omega \right)} \end{array}$$ (1)

where A(ω) and θ(ω) represent the amplitude and phase, respectively, and $-\theta^{\prime }\left(\omega \right)$ denotes the GD. When this signal is characterized in the TF domain, its idealized TF expression is as follows:


$$\begin{array}{c} ITFR\left(t,\omega \right)=A\left(\omega \right)\cdot e^{i\cdot \theta \left(\omega \right)}\cdot \delta (t+\theta^{\prime }(\omega )) \end{array}$$ (2)

where δ(•) denotes the Dirac function. Equation (2) indicates that, under ideal conditions, the signal energy is predominantly concentrated along the GD curve in the TF plane. In practical applications, the TFR of a signal is typically achieved using the short-time Fourier transform (STFT):


$$\begin{array}{c} STFT\left(t,\omega \right)=(2\pi {)}^{-1}\int_{-\infty }^{\infty }\tilde{s}\left(\tau \right)\cdot \tilde{g}(\tau -\omega )\cdot e^{i(\tau -\omega )t}d\tau \end{array}$$ (3)

where $\tilde{g}(•)$ denotes a frequency-domain window function.

The TF characteristics of STFT are further investigated by assuming that the analyzed signal exhibits a slowly varying instantaneous frequency. Specifically, given a sufficiently small value ε, for ∀ω: $\left|A^{\prime }\left(\omega \right)\right|\leq \varepsilon$, $\left|\theta^{\prime \prime }\left(\omega \right)\right|\leq \varepsilon$. Consequently, the signal $\tilde{s}\left(\tau \right)$ in Equation (3) can be approximated as follows:


$$\begin{array}{c} \tilde{s}\left(\tau \right)=A\left(\omega \right)e^{i\left(\theta \left(\omega )+\theta^{\prime }(\omega \right)\left(\tau -\omega \right)\right)} \end{array}$$ (4)

Substituting Equation (4) into Equation (3), the corresponding STFT expression is obtained as follows:


$$\begin{array}{c} STFT\left(t,\omega \right)=A\left(\omega \right)\cdot e^{i\cdot \theta \left(\omega \right)}\cdot g(t+\theta^{\prime }(\omega )) \end{array}$$ (5)

where g(•) represents a time-domain window function. According to Equation (5), the TF energy distribution is spread around the GD curve. To achieve higher TF energy concentration of the feature components, TMSST constructs an estimation operator based on the Dirac function δ(•) to squeeze the TF coefficients of the features onto the GD trajectory:


$$\begin{array}{c} TS\left(\mu ,\omega \right)=\int_{-\infty }^{\infty }STFT\left(t,\omega \right)\cdot \delta (\mu -t_{\text{0}}(t,\omega ))dt \end{array}$$ (6)

After the squeezing operation, although the energy dispersion of the features can be alleviated to some extent, the complex variations in practical signals often prevent them from fully satisfying the assumption of being slowly varying frequency signals. Consequently, errors typically persist in the estimation of the GD for real signals. To achieve more accurate results, TMSST further introduces a multi-iteration mechanism. In practice, the iteration number N is determined according to the convergence of two successive GD estimates. Specifically, the iteration is terminated when the following criterion is satisfied:


$$\begin{array}{c} \iint \left|t_{\text{0}}^{\left[N\right]}\left(t,\omega \right)-t_{\text{0}}^{\left[N-1\right]}\left(t,\omega \right)\right|dt\text{\textbackslash{}hspace\{0.17em}\}d\omega /L^{\text{2}}<\xi \end{array}$$ (7)

where L denotes the signal length and ξ is a prescribed convergence tolerance. Accordingly, the smallest N satisfying this criterion is selected as the iteration number, thereby avoiding unnecessary iterations while maintaining sufficient GD estimation accuracy. As N increases, the estimated GD progressively approaches the true GD, and when N is sufficiently large, it asymptotically converges to the true value:


$$\begin{array}{c} \underset{N\to \infty }{lim}{t_{\text{0}}}^{\left[N\right]}\left(t,\omega \right)=-\theta^{\prime }\left(\omega \right) \end{array}$$ (8)

Based on this concept, the mathematical formulation of TMSST can be derived as follows:


$$\begin{array}{c} \begin{array}{c} MS\left(t,\omega \right)=\int_{-\infty }^{\infty }STFT\left(t,\omega \right)\cdot \delta (\mu -t_{\text{0}}^{\left[N\right]}(\mu ,\omega ))d\mu \\ \text{with }t_{\text{0}}^{\left[N\right]}\left(\mu ,\omega \right)=t_{\text{0}}\left(t_{\text{0}}^{\left[N-1\right]}\left(\mu ,\omega \right),\omega \right) \end{array} \end{array}$$ (9)

### 2.2 Theory of CSA-TMSST

In general, an actual signal can be represented as the superposition of the informative components and background noise, which can be written as:


$$\begin{array}{c} s\left(t\right)=s_{F}\left(t\right)+n\left(t\right) \end{array}$$ (10)

where s(t) represents the raw signal and $s_{F}\left(t\right)$ denotes the fault-related components. n(t) denotes the background noise. The estimation of the feature component $s_{F}\left(t\right)$ can be formulated as a feature coefficient estimation problem in a sparse linear transform space, as follows:


$$\begin{array}{c} s_{F}\left(t\right)=A\cdot c \end{array}$$ (11)

where A is the linear transformation matrix, for which the Fourier transform (FT) is employed in this study, and c represents the feature coefficients under this linear transformation. The optimal estimation of c is typically obtained by solving a classical least squares model:


$$\begin{array}{c} \underset{c}{min}\left\{F\left(c)=\frac{\text{1}}{\text{2}}{\|s-A\cdot c\|}_{\text{2}}^{\text{2}}+\lambda \cdot \psi (c\right)\right\} \end{array}$$ (12)

where ψ(c) denotes the sparsity penalty function. This work introduces a standard non-convex sparsity penalty function (the GMC penalty), which is defined as follows:


$$\begin{array}{c} \psi_{\text{GMC}}\left(c\right)={\|c\|}_{\text{1}}-\underset{v}{min}\{{\|v\|}_{\text{1}}+\frac{\gamma }{2\lambda }{\|A(c-v)\|}_{\text{2}}^{\text{2}}\} \end{array}$$ (13)

where γ is the control parameter of the GMC penalty. To maintain the convexity of the GMC-regularized objective function and ensure stable optimization, γ is restricted to 0<γ≤1 [30]. The λ denotes the regularization parameter that controls the trade-off between data fidelity and sparsity. An excessively small λ may result in insufficient noise suppression, whereas an excessively large λ may attenuate weak fault-related coefficients. In this study, λ is empirically selected through preliminary tests to achieve an appropriate balance between background noise suppression and the preservation of weak fault-related impulsive components. Based on Equation (13), the optimal estimate of the coefficient c in Equation (12) can be reformulated as:


$$\begin{array}{c} \underset{c}{min}\left\{F\left(c)=\frac{\text{1}}{\text{2}}{\|s-A\cdot c\|}_{\text{2}}^{\text{2}}+\lambda \cdot \psi_{\text{GMC}}(c\right)\right\} \end{array}$$ (14)

Upon obtaining the coefficient c, the signal $s_{F}\left(t\right)$ can be derived via Equation (11). This resulting signal represents the preliminary denoised output processed by the CSA. Furthermore, by applying TMSST to the signal $s_{F}\left(t\right)$, the final TFR is given by:


$$\begin{array}{c} \begin{array}{c} MS_{s_{F}}\left(\mu ,\omega \right)\\ =\int_{-\infty }^{\infty }STFT_{s_{F}}\left(t,\omega \right)\cdot \delta_{s_{F}}(\mu -t_{\text{0}}^{\left[N\right]}(\mu ,\omega ))dt \end{array} \end{array}$$ (15)

To further extract fault-related impulsive components, a binary TF mask (TFM) is constructed according to the distribution characteristics of periodic fault impulses in the TMSST representation. Considering that bearing fault impulses generally exhibit broadband resonance characteristics, the prominence of periodic impulsive information varies across different frequency locations. Therefore, following the impulse feature extraction strategy of TMSST, the TF envelope spectrum (TFES) is first calculated at each frequency location to quantify the prominence of periodic impulsive features. The TFES is defined as follows:


$$\begin{array}{c} TFES\left(\omega \right)=max\left|\int_{-\infty }^{\infty }\left(\;\left|MS_{s_{F}}\left(t,\omega \right)\right|-\phi \left(\omega \right)\right)e^{-j\xi t}\text{\textbackslash{}hspace\{0.17em}\}dt\right| \end{array}$$ (16)

where ϕ(ω) is the mean value of $MS_{s_{F}}\left(t,\omega \right)$ at frequency ω. The value of TFES(ω) reflects the prominence of periodic impulsive features at the corresponding frequency location. A larger value indicates that the frequency location contains more pronounced fault-related periodic impulsive information. Based on the TFES, the binary mask in the frequency direction $TFM_{F}\left(t,\omega \right)$ is defined as follows:


$$\begin{array}{c} TFM_{F}\left(t,\omega \right)=\left\{ \begin{array}{cc} @ll@1, & \;TFES\left(\omega \right)>\alpha \\ 0, & \;otherwise \end{array}\right. \end{array}$$ (17)

where α represents the mean value of the TFES over all frequency bins. Based on this threshold, $TFM_{F}\left(t,\omega \right)$ retains the frequency regions containing prominent fault-related periodic impulsive features while suppressing frequency components unrelated to the fault. Next, the frequency selected TMSST amplitudes are integrated along the frequency direction to characterize the temporal distribution of transient impulses, and the impulse indicator I(t,ω) is defined as follows:


$$\begin{array}{c} I\left(t,\omega \right)=\int_{-\infty }^{\infty }\;TFM_{F}\left(t,\omega \right)\left|\;MS_{s_{F}}\left(t,\omega \right)\right|\text{\textbackslash{}hspace\{0.17em}\}d\omega \end{array}$$ (18)

where the impulse indicator I(t,ω) characterizes the temporal distribution of fault-related impulsive energy, and its magnitude reflects the prominence of the transient impulsive response. A larger value of I(t,ω) indicates a stronger fault-related transient impulse at the corresponding time instant. Based on the distribution of I(t,ω), the binary mask in the time direction $TFM_{T}\left(t,\omega \right)$ is defined as follows:


$$\begin{array}{c} TFM_{T}\left(t,\omega \right)=\left\{ \begin{array}{cc} @ll@1, & \;I\left(t,\omega \right)>\beta \\ 0, & otherwise \end{array}\right. \end{array}$$ (19)

where β denotes the mean value of the impulse indicator I(t,ω) over the analyzed time interval. This threshold is used to retain the time instants associated with prominent transient impulses while suppressing the background noise between successive fault induced impulses. Finally, the binary TFM(t,ω) is constructed as follows:


$$\begin{array}{c} TFM\left(t,\omega \right)=TFM_{T}\left(t,\omega \right)\cdot TFM_{F}\left(t,\omega \right) \end{array}$$ (20)

Therefore, only the TMSST coefficients that simultaneously satisfy the frequency-domain impulsiveness criterion and the time-domain transient criterion are retained for subsequent reconstruction. Otherwise, they are regarded as noise or irrelevant interference and suppressed. On this basis, the TMSST coefficients retained after TFM-based selection are reconstructed to obtain the fault-related impulsive signal $s_{R}\left(t\right)$, which is expressed as follows:


$$\begin{array}{c} s_{R}(t)=(2\pi \tilde{g}(0){)}^{-1}\cdot \int_{-\infty }^{\infty }\int_{-\infty }^{\infty }MS_{s_{F}}\left(\mu ,\omega \right)\cdot TFM\left(t,\omega \right)\cdot e^{i\omega t}d\mu d\omega \end{array}$$ (21)

where $MS_{s_{F}}\left(t,\omega \right)$ denotes the TMSST coefficients of the GMC-enhanced signal $s_{F}\left(t\right)$. Based on the above theoretical analysis, CSA-TMSST first employs GMC to sparsely enhance and reconstruct the fault-related transient impulsive components from the raw noisy signal, thereby reducing the influence of strong background noise on the subsequent TFA. TMSST is then applied to further improve the concentration of transient impulsive energy in the TF plane through iterative time reassignment. In addition, TFES and TFM are employed to select and reconstruct the fault-related TF coefficients. Finally, the reconstructed signal $s_{R}\left(t\right)$ containing the dominant fault-related impulsive features is obtained. The main implementation procedure of the CSA-TMSST method is shown in Algorithm 1.

**Algorithm 1:** Implementation of CSA-TMSST

**Step 1: Initialization**

Choose the vibration signal x(t);

Choose the GMC parameters λ and γ;

Choose the FT-based transformation matrix A;

**Step 2: Sparse enhancement**

Calculate c according to Eq. (14);

Calculate $s_{F}\left(t\right)$ using the matrix A;

Calculate $s_{F}\left(t\right)\leftarrow Ac$;

**Step 3: Iterative time reassignment**

Calculate the $STFT_{S_{F}\left(\mu ,\omega \right)}$ of $s_{F}\left(t\right)$;

Initialize ${t_{\text{0}}}^{\left[0\right]}\left(\mu ,\omega \right)$;

k←1;

**repeat**

 Update ${t_{\text{0}}}^{\left[k\right]}\left(\mu ,\omega \right)$;

 Calculate the ${t_{\text{0}}}^{\left[k\right]}\left(\mu ,\omega \right)$; ${t_{\text{0}}}^{\left[k-1\right]}\left(\mu ,\omega \right)$;

 k←k+1;

**until** Eq. (7) is satisfied;

N←k−1;

Calculate $MS_{s_{F}}\left(\mu ,\omega \right)$

**Step 4: Reconstruction**

$TFM_{F}\leftarrow 0$; $TFM_{T}\leftarrow 0$;

**for** j=1:J

 Calculate $TFES\left(\omega_{j}\right)$ according to Eq. (16);

**end for**

 $\alpha \leftarrow \frac{\text{1}}{J}\sum_{j=1}^{J}TFES\left(\omega_{j}\right)$;

**for** j=1:J

 **if** $TFES\left(\omega_{j}\right)>\alpha$

  $TFM_{F}\left(\mu ,\omega_{j}\right)\leftarrow 1;$

 **end if**

**end for**

**for** i=1:N

 $I\left(\mu_{i}\right)\leftarrow \sum_{j=1}^{J}TFM_{F}\left(\mu_{i},\omega_{j}\right)\left|\;MS_{s_{F}}\left(\mu_{i},\omega_{j}\right)\right|\text{\textbackslash{}hspace\{0.17em}\Delta \omega \}$;

**end for**

  $\beta \leftarrow \frac{\text{1}}{N}\sum_{i=1}^{N}I\left(\mu_{i}\right)$;

**for** i=1:N

 **for** j=1:J

  **if** $I\left(\mu_{i}\right)>\beta$

   $TFM_{T}\left(\mu_{i},\omega_{j}\right)\leftarrow 1$;

  **end if**

   $TFM\left(\mu_{i},\omega_{j}\right)\leftarrow TFM_{T}\left(\mu_{i},\omega_{j}\right)\cdot TFM_{F}\left(\mu_{i},\omega_{j}\right)$

 **end for**

**end for**

Calculate $s_{R}\left(t\right)$ according to Eq. (21);

**output** $s_{R}\left(t\right)$

### 2.3 Bearing fault diagnosis method via CSA-TMSST

Fig 1 presents the flowchart of the CSA-TMSST method applied to bearing fault diagnosis. On the basis of the theoretical foundations established in Sections 2.1 and 2.2, the implementation details of the CSA-TMSST for bearing fault diagnosis are presented as follows.

**Fig 1:**
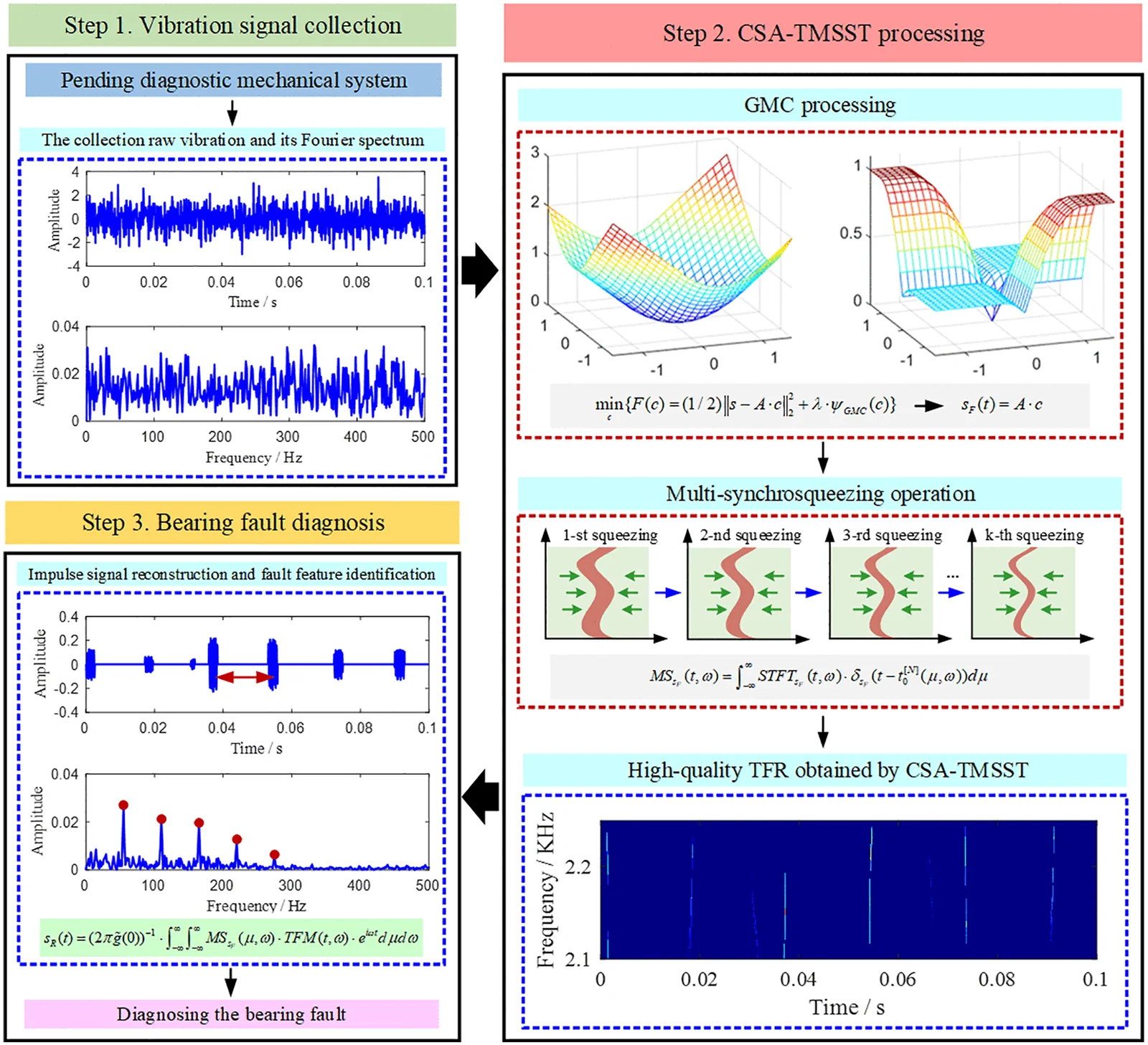
Fault diagnosis using CSA-TMSST.

(i). Step 1. Acquisition and preprocessing of raw bearing vibration signals. The raw signal is processed via CSA to obtain the pre-denoised signal $s_{F}\left(t\right)$.
(ii). Step 2. TFR and fault impulse signal reconstruction. TMSST is employed for processing the CSA-preprocessed signal $s_{F}\left(t\right)$, yielding a high-quality TFR. Subsequently, the fault-related impulse signal $s_{R}\left(t\right)$ is extracted and reconstructed.
(iii). Step 3. Fault identification and diagnosis. The extracted impulsive component is analyzed via envelope spectral analysis, and bearing faults are effectively identified and diagnosed based on the characteristic frequency components in the resulting envelope spectrum.

## 3 Numerical analysis

To preliminarily validate the effectiveness of CSA-TMSST in enhancing fault features and diagnosing bearing faults, a simulated outer race fault model of a rolling bearing is established, whose mathematical expression is given as follows:


$$\begin{array}{c} \begin{array}{c} s(t)=\sum_{i=0}^{I}[A_{\text{0}}cos(2\pi f_{m}t)+1]\cdot e^{-B(t-iT_{\text{0}}-\tau_{i})}\;u\text{(}t-iT_{\text{0}}-\tau_{i}\text{)}\cdot cos\left[2\pi f_{n}\left(t-iT_{\text{0}}-\tau_{i}\right)\right]+n\left(t\right) \end{array} \end{array}$$ (22)

where $A_{\text{0}}$ and $f_{m}$ represent the resonance intensity and modulation frequency, respectively. B denotes the system damping coefficient, and $f_{n}$ is the natural resonance frequency of the system. Additionally, $T_{\text{0}}=1/f_{\text{0}}$ is the fault impulse period, $\tau_{i}$ is the time delay, and n(t) is the noise interference. The function u(·) denotes the Heaviside unit step function, which ensures the causality of each impulse response.

The simulated signal is generated with a sampling frequency of $f_{s}=10\text{kHz}$. The key parameters include a resonance intensity of $A_{\text{0}}=2$, modulation frequency of $f_{m}=15\text{Hz}$, damping coefficient of B=800, and natural resonance frequency $f_{n}=2200\text{Hz}$. Under the given operating conditions, the outer race fault characteristic frequency is determined to be $f_{\text{0}}=55\text{Hz}$. Furthermore, Gaussian white noise n(t) is added to the simulated signal with a signal-to-noise ratio (SNR) of −10 dB to simulate a strong noise environment.

Fig 2(a) presents the simulated outer race fault signal under strong noise interference. The fault-related impulsive features are largely submerged in the noise background, rendering the periodic impact components difficult to identify directly from the time-domain representation. In Fig 2(b), the fault characteristic frequency and its harmonic components are difficult to identify. To evaluate the effectiveness of the CSA-TMSST method in weak fault feature enhancement and extraction, EMD, VMD, CSA, TMSST, and L1-TMSST are used as comparative methods. For EMD, the first six IMFs are retained for analysis because the subsequent IMFs mainly contain low frequency residual components and contribute little fault-related information. For VMD, the number of modes is empirically set to 6, and the penalty parameter is set to 2000. Using six analyzed components for both decomposition methods facilitate a consistent comparison of their fault-feature extraction performance. For consistency, the same EMD component-selection strategy and VMD parameter settings are adopted in the two subsequent experimental cases.

**Fig 2:**
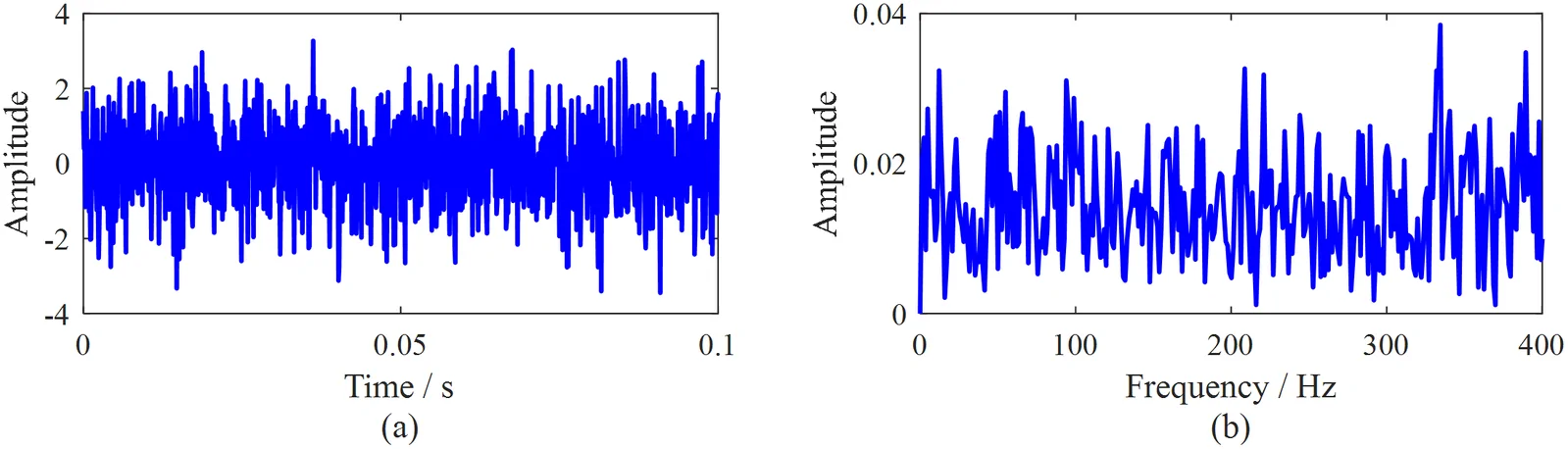
Waveform and envelope spectrum of the simulated outer race fault signal: (a) time-domain waveform; (b) envelope spectrum.

Fig 3 illustrates six IMFs obtained via EMD and their envelope spectrum analysis results. In Fig 3(a), EMD is prone to mode mixing when processing complex bearing fault signals, causing the extracted transient impulsive components to fail to clearly characterize the periodic impulsive features. Meanwhile, Fig 3(b) indicates that the envelope spectra of each IMF component fail to clearly identify the fault characteristic frequency.

**Fig 3:**
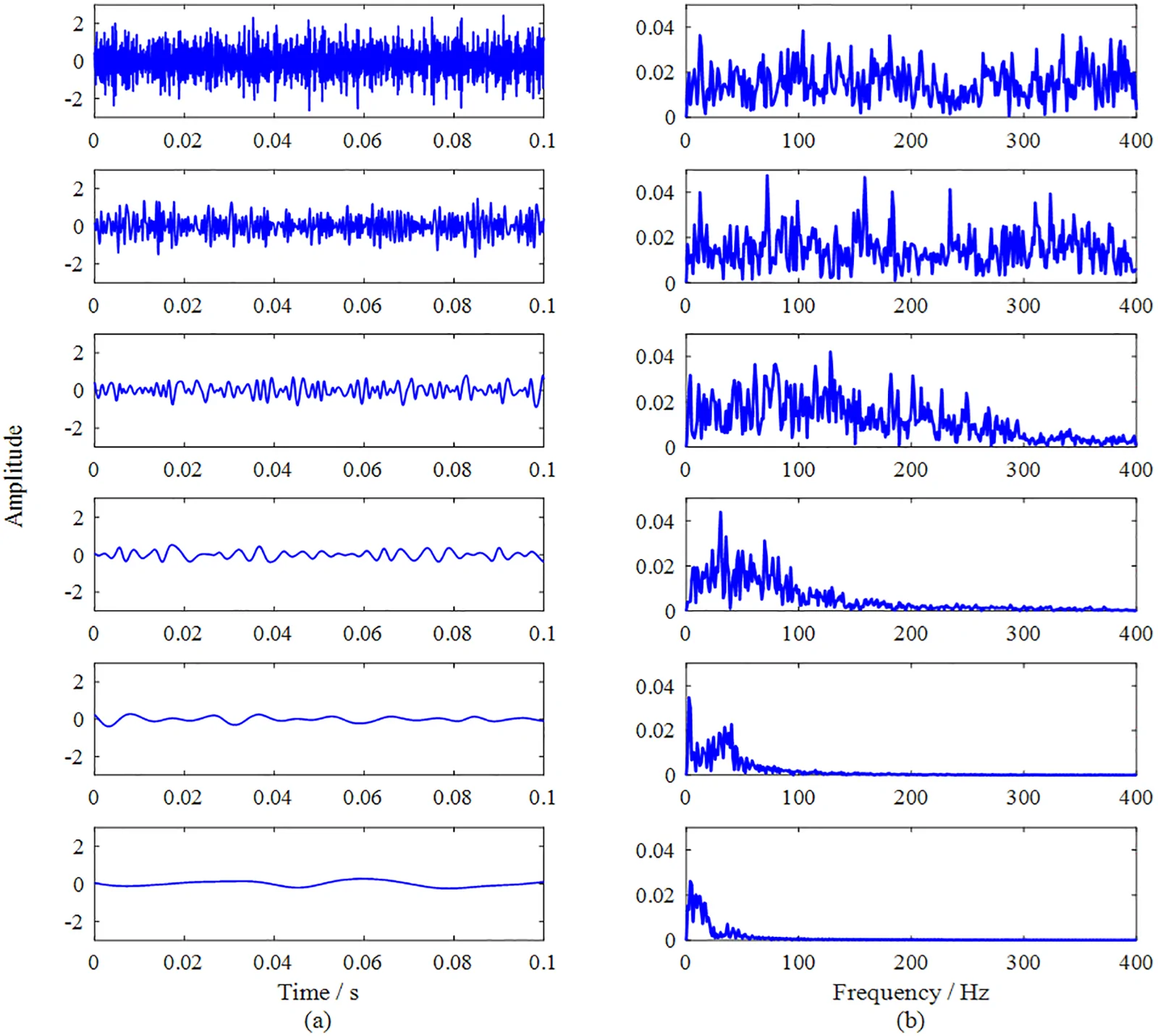
EMD decomposition results of the simulated signal: (a) IMFs; (b) corresponding envelope spectra.

Fig 4(a) and (b) present the six modes obtained using the VMD method and their corresponding envelope spectrum results. Although VMD can alleviate the mode mixing problem to some extent, periodic impulsive components are still not effectively separated under strong noise conditions, thereby affecting the accurate identification of the fault characteristic frequency.

**Fig 4:**
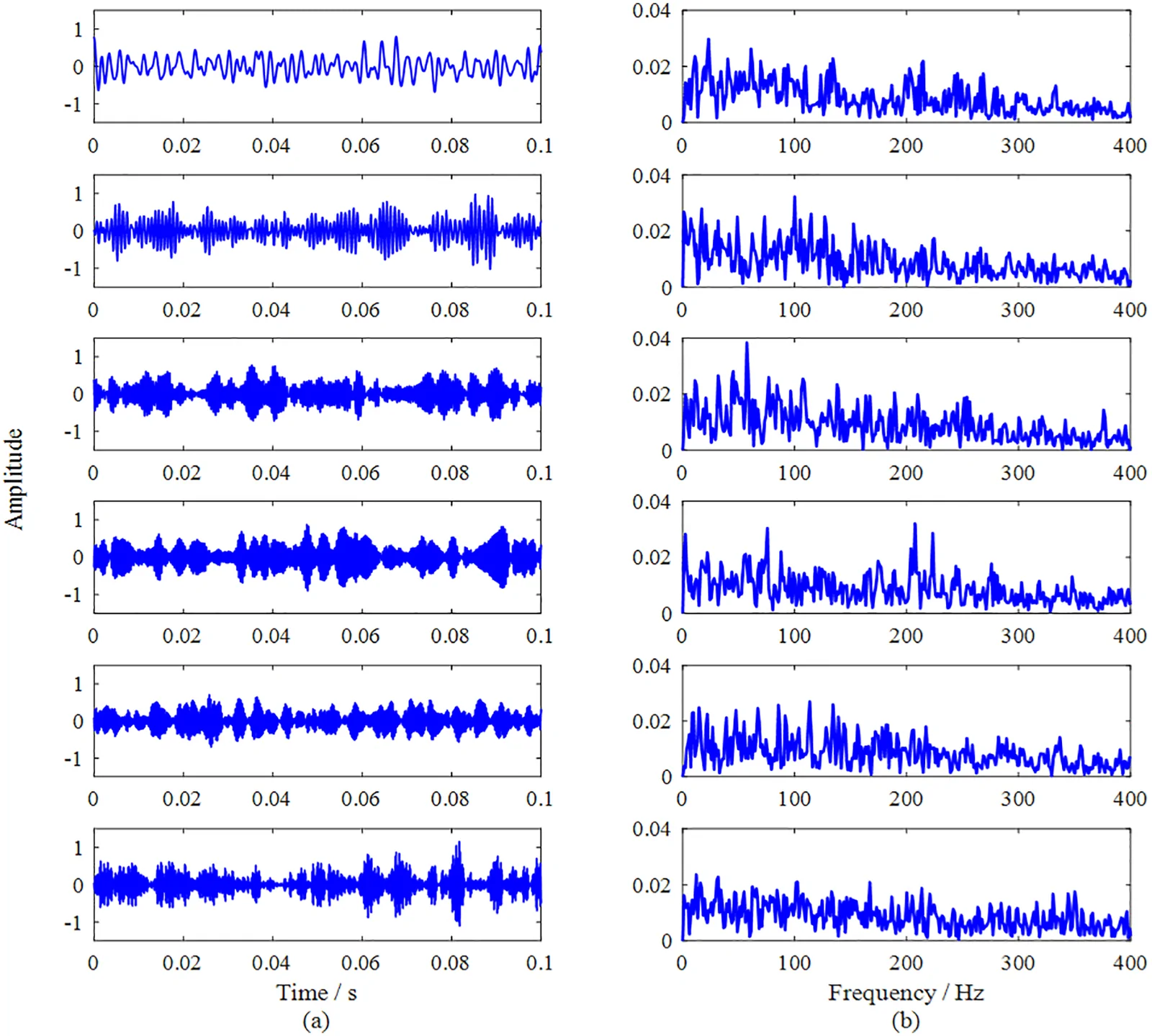
VMD decomposition results of the simulated signal: (a) decomposed modes; (b) corresponding envelope spectra.

Fig 5 presents the results obtained by applying CSA to the simulated outer race fault signal. Fig 5(a) shows that CSA suppresses part of the broadband background noise and preserves a portion of the fault-related transient components. However, considerable residual noise remains in the reconstructed signal, making the periodic impulsive structure difficult to identify directly. Fig 5(b) exhibits a prominent fault characteristic peak at approximately 54.9 Hz, together with its harmonic components, indicating that the GMC-based sparse reconstruction enhances weak fault-related information to a certain extent. Nevertheless, some non-fault-related components and residual noise are still present in the spectrum. These results demonstrate that CSA provides preliminary sparse enhancement of weak impulsive components. However, CSA alone remains limited in suppressing strong noise and enhancing periodic fault features.

**Fig 5:**
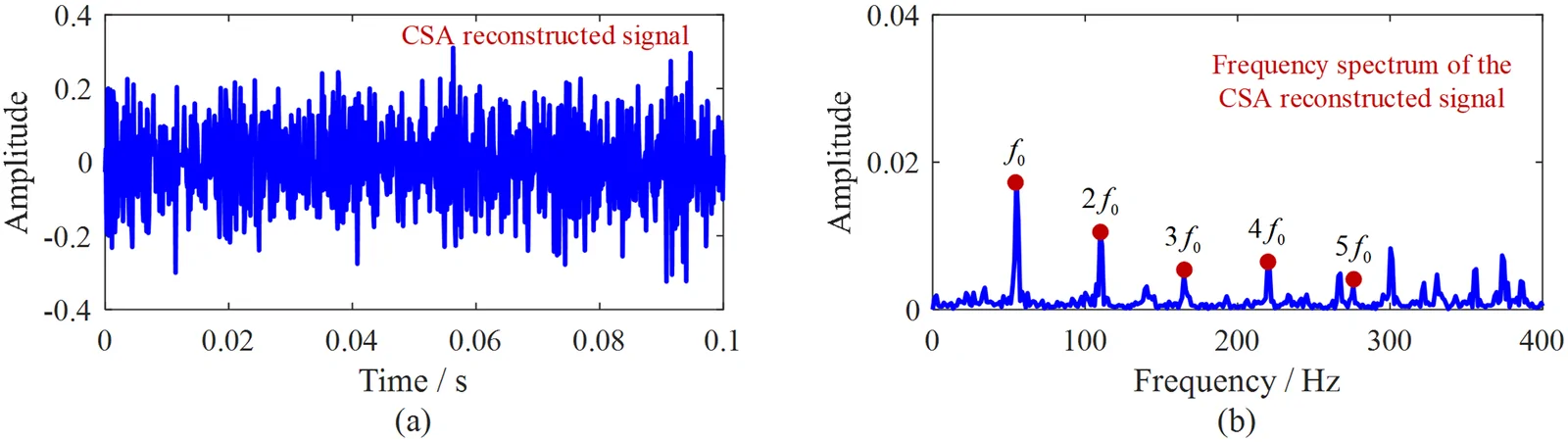
CSA processing results of the simulated signal: (a) reconstructed signal; (b) envelope spectrum of the reconstructed signal.

Fig 6 shows the results obtained by directly applying TMSST to the simulated outer race fault signal. In Fig 6(a), strong noise leads to a dispersed TF energy distribution, resulting in the fault impulsive energy failing to be effectively concentrated. Consequently, the extracted impulsive signal in Fig 6(b) suffers from missing fault features and fails to exhibit obvious periodic fault characteristics. This further degrades the envelope spectrum in Fig 6(c), making the outer race fault characteristic frequency and its harmonic components difficult to distinguish accurately.

**Fig 6:**
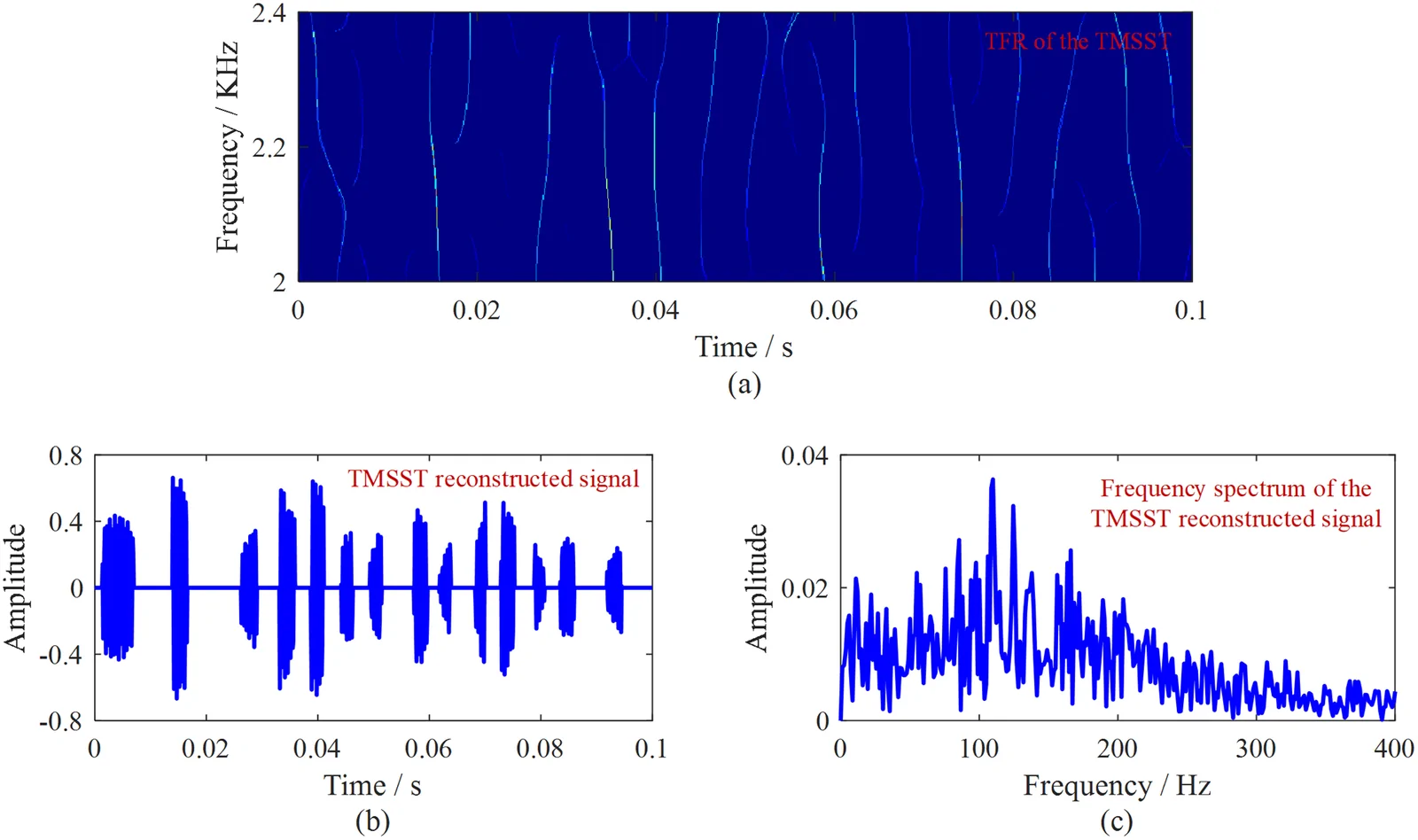
TMSST analysis results of the simulated fault signal: (a) TFR obtained by TMSST; (b) reconstructed fault-related impulsive component; (c) envelope spectrum.

Figs 7(b) and 8(b) further show that both methods successfully reconstruct periodically occurring fault impulses. Nevertheless, the impulsive components reconstructed by CSA-TMSST exhibit more clearly defined temporal locations and better preservation of the impulse amplitudes. The interval between two adjacent impacts is $T_{\text{0}}\approx 0.0183\text{s}$, corresponding to a fault characteristic frequency of $f_{\text{0}}\approx 54.6\text{Hz}$, which agrees well with the preset outer race fault frequency. In Figs 7(c) and 8(c), the characteristic frequency $f_{\text{0}}$ and its multiple harmonics can be identified by both methods. However, the spectral peaks obtained by CSA-TMSST are more prominent, while the residual spectral background and non-fault-related components are further suppressed. In comparison, the fault-related spectral peaks obtained by L1-TMSST exhibit lower amplitudes. This difference indicates that the GMC-based non-convex sparse model provides improved preservation of fault-related transients.

**Fig 7:**
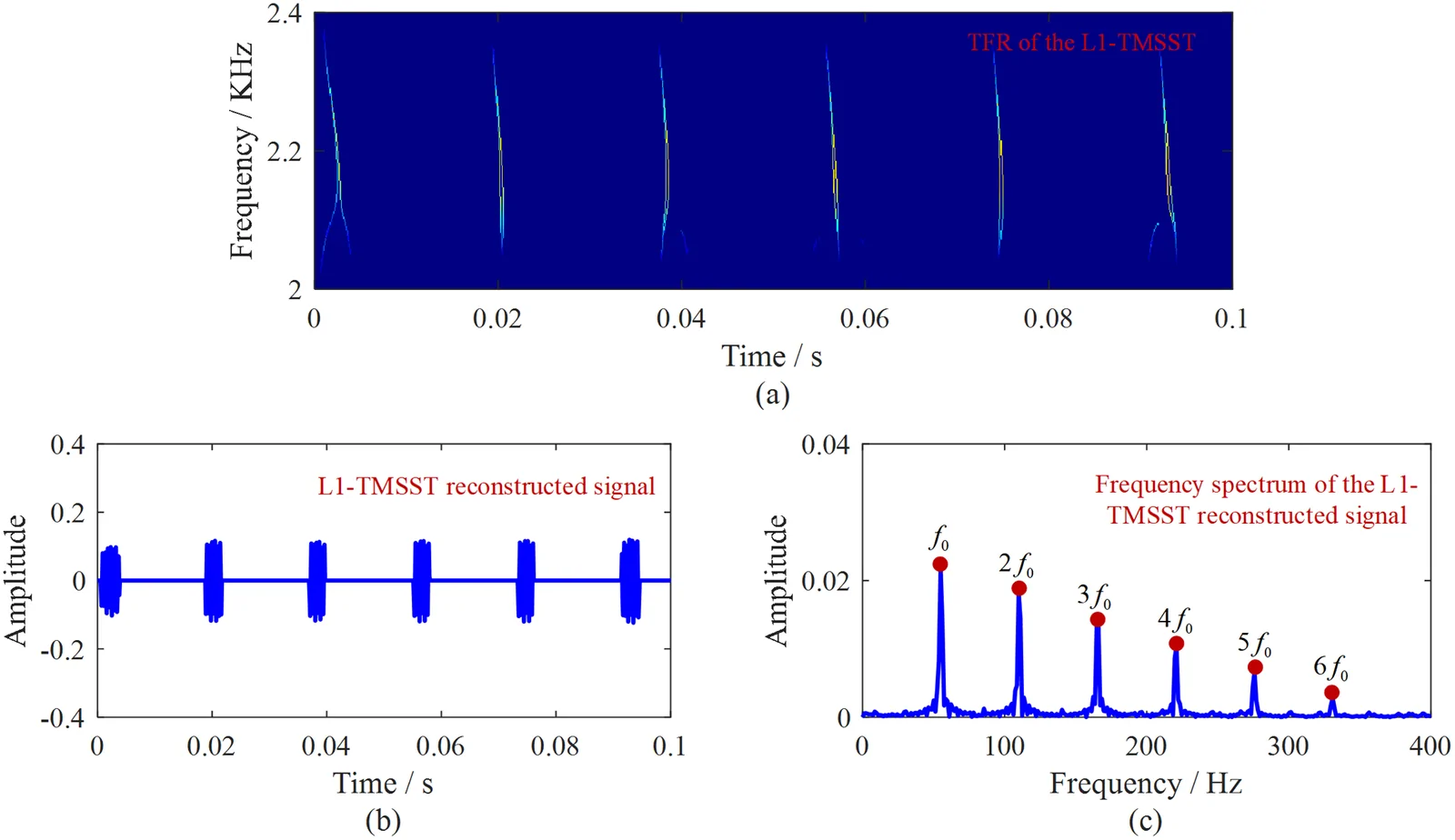
L1-TMSST analysis results of the simulated fault signal: (a) TFR obtained by L1-TMSST; (b) reconstructed fault-related impulsive component; (c) envelope spectrum.

**Fig 8:**
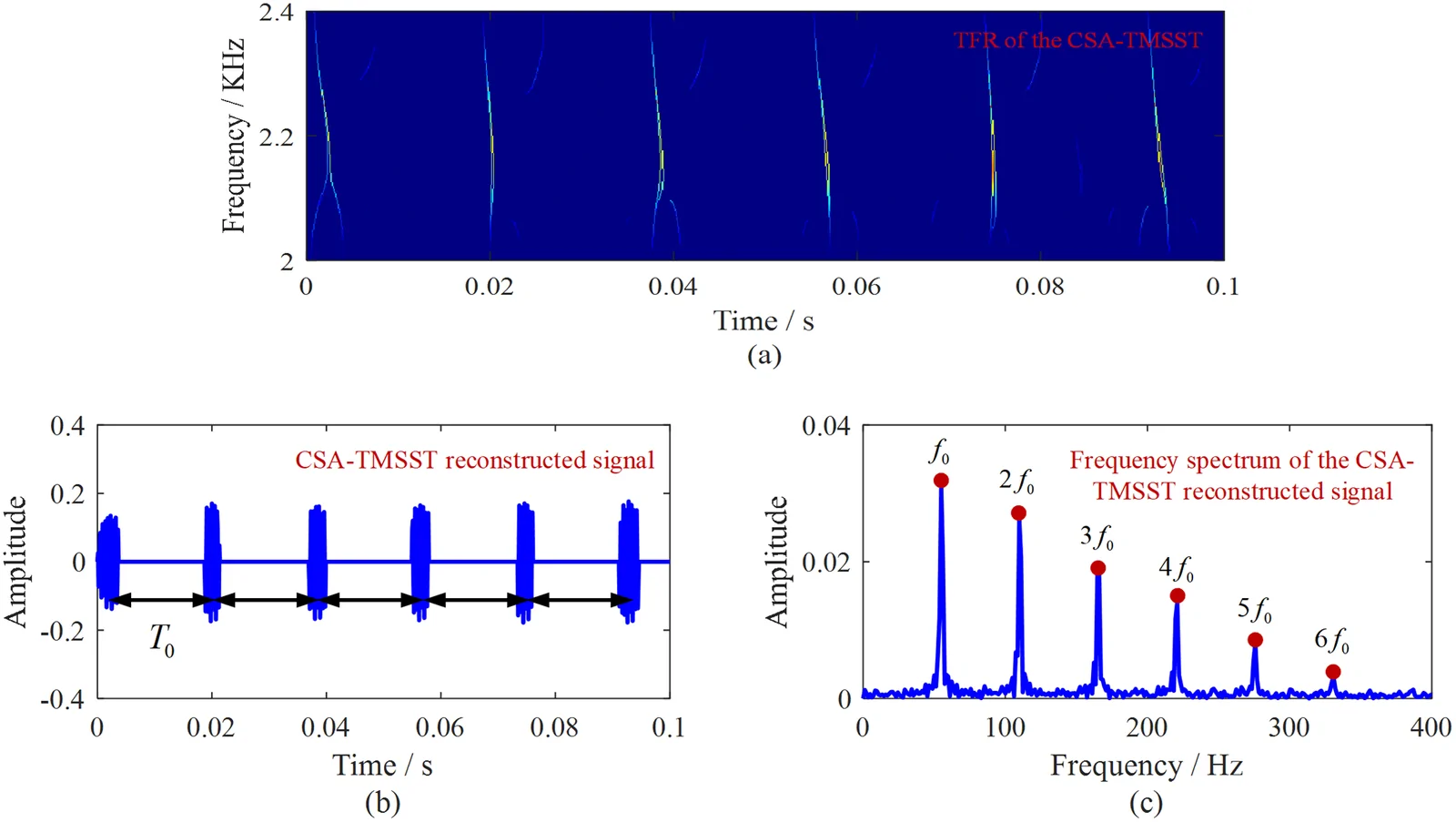
CSA-TMSST analysis results of the simulated fault signal: (a) TFR obtained by CSA-TMSST; (b) reconstructed fault-related impulsive component; (c) envelope spectrum.

To quantitatively evaluate the noise robustness and signal reconstruction performance of the proposed method, Gaussian white noise at different levels was added to the simulated signal. The input SNR was varied from −10 dB to 0 dB. The output SNR and root-mean-square error (RMSE) were employed as quantitative evaluation metrics. A higher output SNR indicates better noise suppression and fault signal reconstruction performance, whereas a lower RMSE indicates a smaller deviation between the reconstructed signal and the clean reference signal.

Fig 9 presents a quantitative comparison of CSA-TMSST, L1-TMSST, CSA, TMSST, VMD, and EMD under different input SNR levels. As the input SNR increases from −10 dB to 0 dB, the output SNR values of all methods generally increase, while the corresponding RMSE values gradually decrease, indicating improved reconstruction performance as the noise intensity decreases. Among the compared methods, CSA-TMSST consistently achieves the highest output SNR and the lowest RMSE over the entire investigated SNR range. In particular, at an input SNR of −10 dB, CSA-TMSST achieves an output SNR of 2.5125 dB and an RMSE of 0.1277, demonstrating that the proposed method can effectively recover weak fault-related impulsive components even under severe noise interference. When the input SNR increases to 0 dB, the output SNR of CSA-TMSST further increases to 7.4581 dB, while the RMSE decreases to 0.0722.

**Fig 9:**
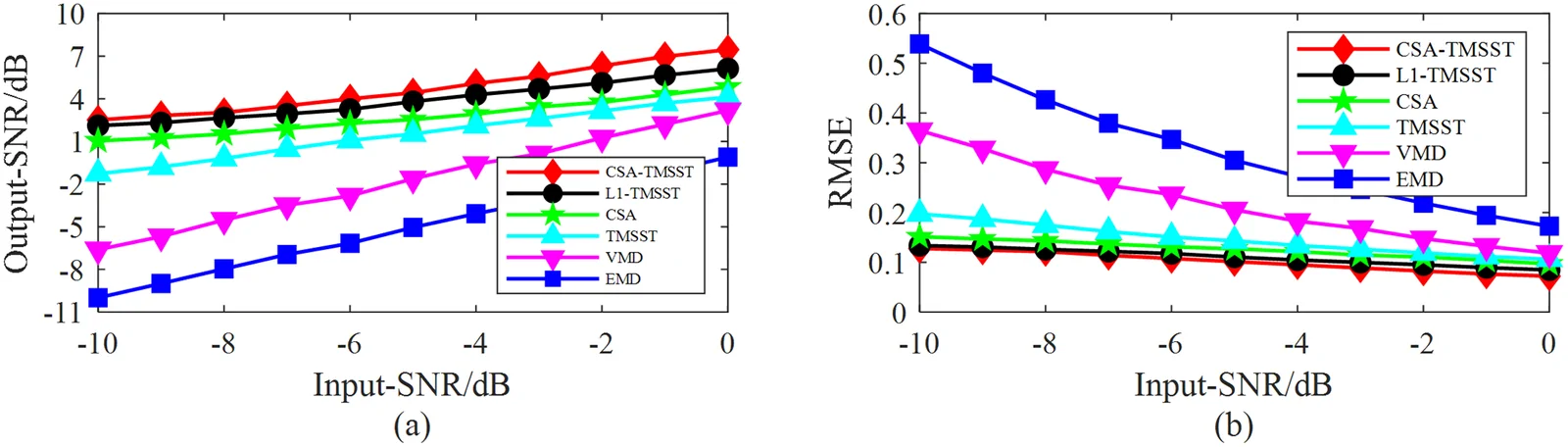
Quantitative performance comparison of different methods under varying input SNR levels: (a) output SNR; (b) RMSE.

Compared with L1-TMSST and CSA, CSA-TMSST exhibits superior signal reconstruction performance. Specifically, GMC-based sparse reconstruction effectively suppresses background noise while preserving weak transient impulses, and the subsequent TMSST further concentrates the fault-related TF energy. In addition, CSA-TMSST significantly outperforms the raw TMSST, VMD, and EMD, particularly at low input SNRs. These results quantitatively demonstrate the effectiveness and robustness of CSA-TMSST for weak fault feature extraction under different noise levels.

## 4 Experimental validations

### 4.1 Verification with laboratory test rig vibration data

To further evaluate the effectiveness of CSA-TMSST, this section conducts experimental validation using a self-built bearing fault diagnosis platform. An outer race fault signal from a rolling bearing is selected for analysis, and the associated experimental platform is shown in Fig 10(a). Fig 10(b) shows the defective bearing used in the fault test. During the experimental test, the drive motor shaft rotated at 1500 rpm, with the test bearing serving as its support. Vibration signals were obtained via an accelerometer mounted on the motor side, with a sampling frequency of 2560 Hz. To replicate the noisy environment of a real industrial scene, the raw signal was contaminated with added noise. Table 1 presents the detailed parameters of the experimental bearing. Based on these values, the theoretical characteristic frequency of the outer race defect is approximately 50 Hz.

**Table 1:** Key geometric characteristics of the experimental bearing.

Outer race diameter	Inner race diameter	Pitch diameter	Roller diameter	Roller count	Contact angle
35 mm	15 mm	25 mm	6 mm	8	0

**Fig 10:**
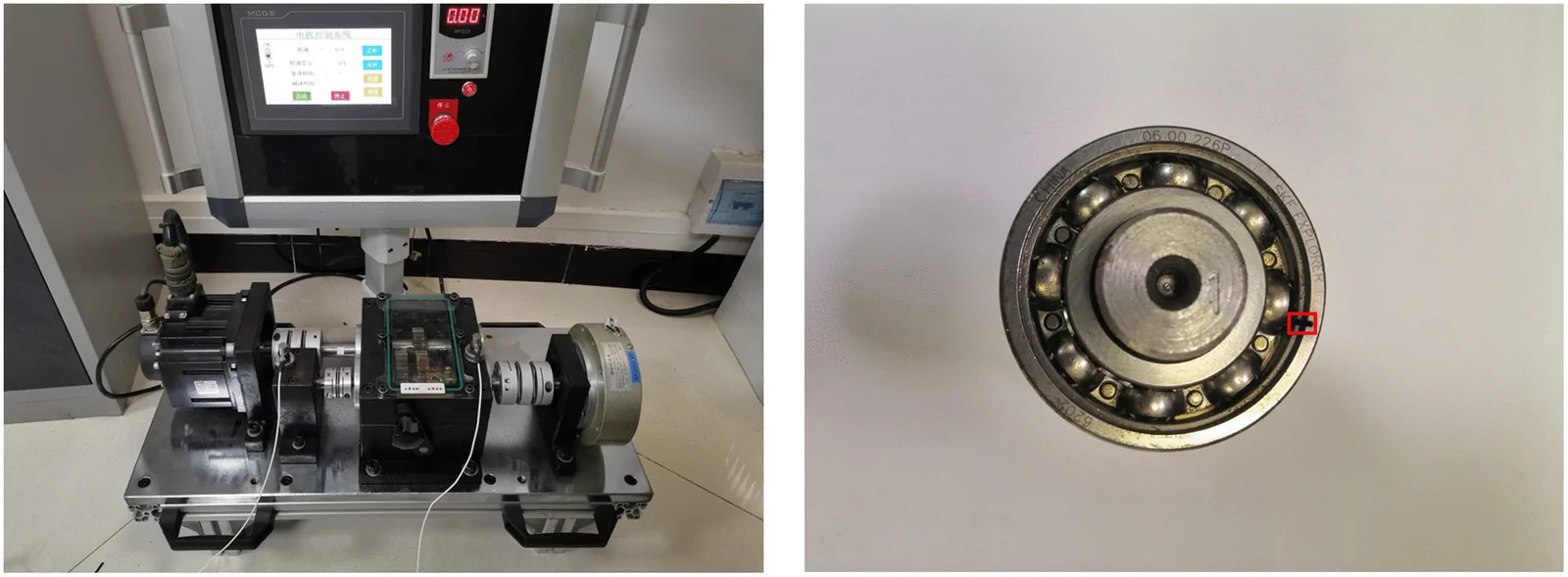
Fault test platform: (a) overall structure of the experimental setup; (b) defective bearing.

As shown in Fig 11(a), strong noise severely masks the fault impulsive features, making the periodic impact components difficult to distinguish in the time-domain signal. Fig 11(b) further indicates that the characteristic frequency corresponding to the outer race defect cannot be clearly identified. Fig 12 presents the analysis results obtained by EMD. Fig 12(a) shows that, under strong noise interference, EMD is prone to mode mixing, causing the six IMFs to fail to effectively reveal the fault-related periodic impulsive components. Furthermore, Fig 12(b) shows that neither the fault frequency nor the harmonic components are clearly distinguishable.

**Fig 11:**
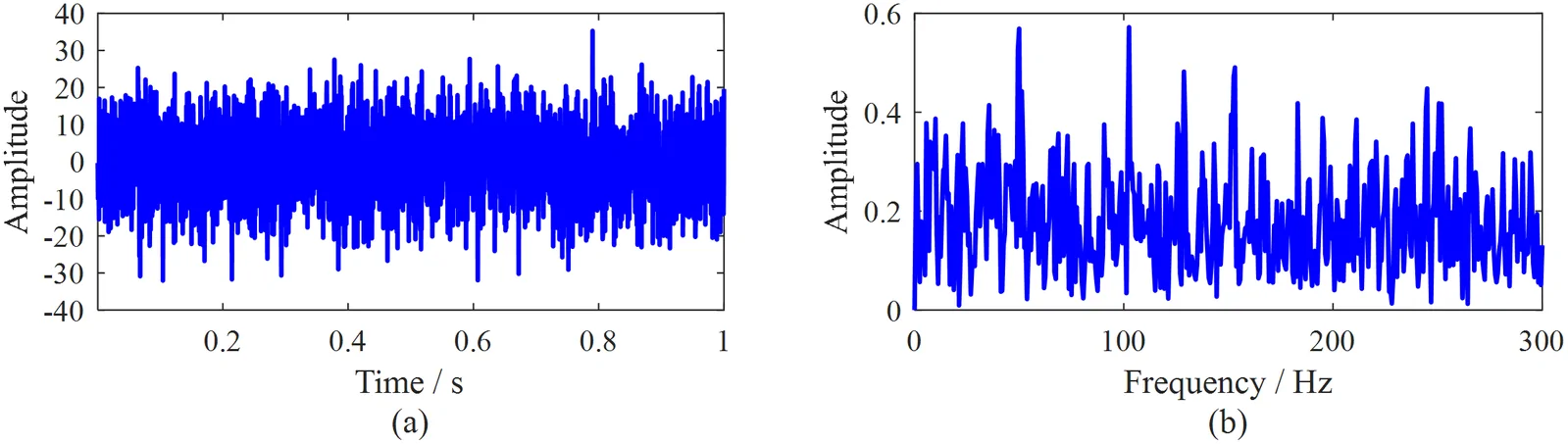
Signal acquired from the test bench: (a) waveform; (b) envelope spectrum.

**Fig 12:**
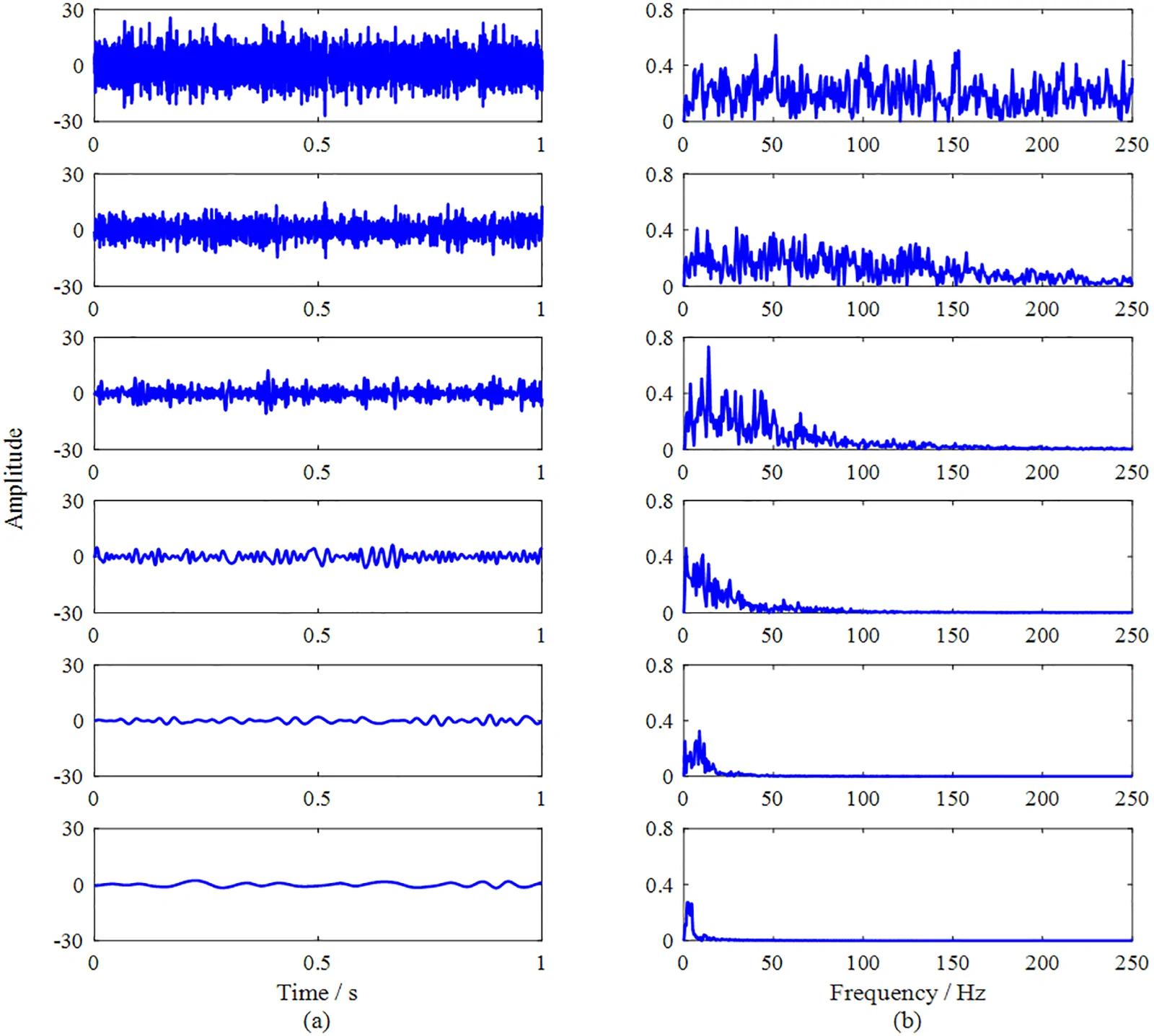
EMD analysis results of the outer race fault signal: (a) IMFs; (b) envelope spectra of the IMFs.

Similarly, Fig 13 presents the VMD decomposition results, consisting of six modes and their envelope spectra. Fig 13(a) and (b) indicate that, although VMD can alleviate the mode mixing problem to some extent, its ability to extract weak periodic impulse components remains limited in the presence of strong noise, making accurate identification of fault-related information challenging.

**Fig 13:**
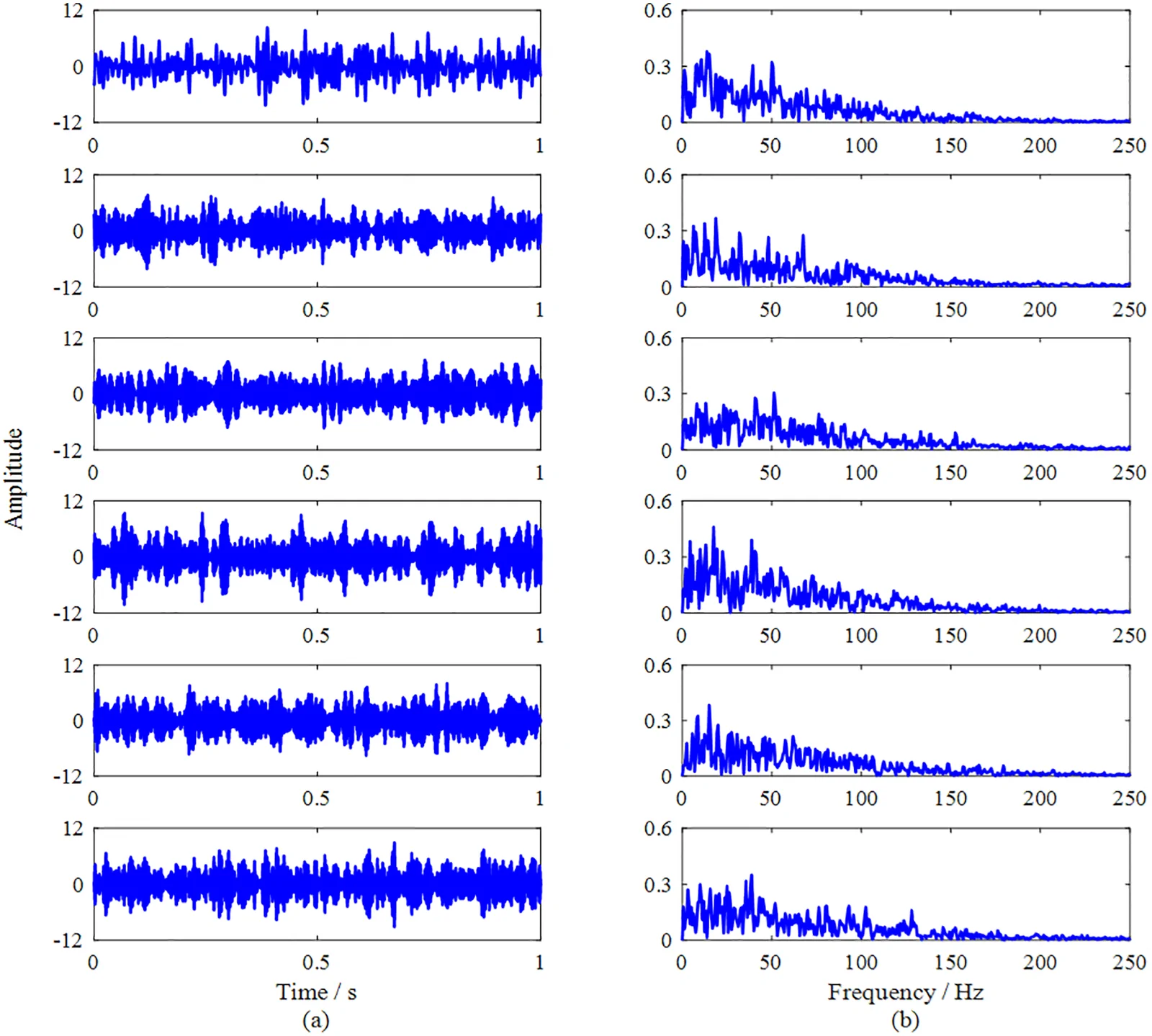
VMD analysis results of the outer race fault signal: (a) decomposed modes; (b) envelope spectra.

Fig 14 presents the results obtained using CSA. Fig 14(a) indicates that the sparse reconstruction suppresses part of the broadband noise and preserves fault-related transient components. However, considerable residual interference remains in the reconstructed signal, making the periodic impulsive pattern difficult to identify directly. In the corresponding envelope spectrum shown in Fig 14(b), a spectral peak near the theoretical outer race fault frequency $f_{\text{0}}\approx 50\text{Hz}$ can be observed, together with several corresponding harmonics, particularly around $3f_{\text{0}}$ and $4f_{\text{0}}$. This indicates that CSA enhances some of the weak fault information obscured in the original signal. Nevertheless, several non-fault-related spectral components remain prominent, while the harmonic structure associated with the fault is incomplete. Therefore, although CSA improves the detectability of the outer race fault frequency, sparse reconstruction alone does not provide sufficiently clear periodic impulsive features under strong noise interference.

**Fig 14:**
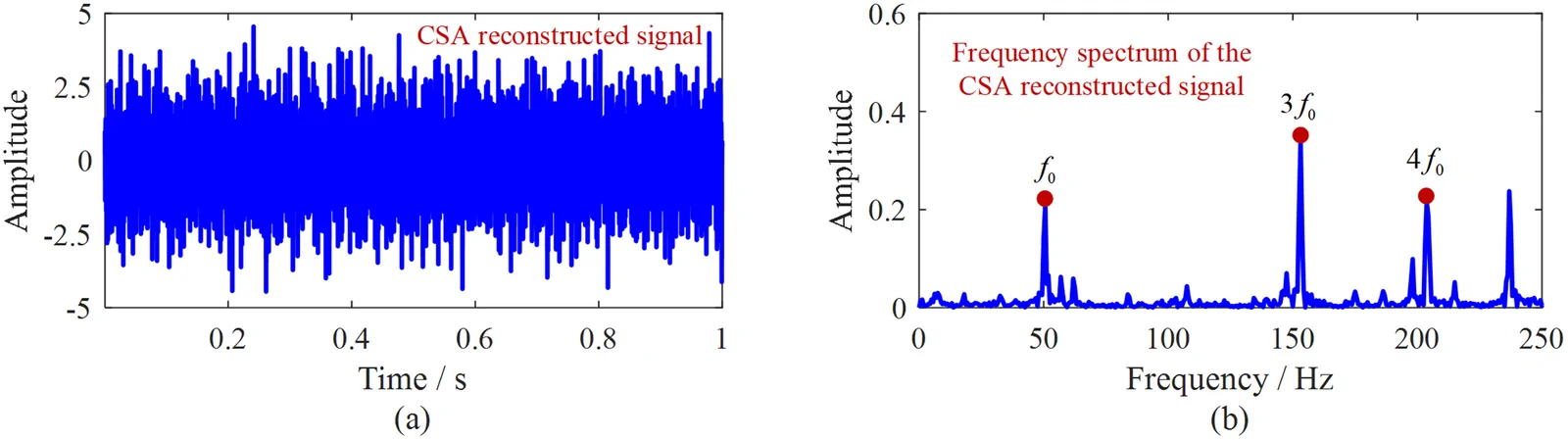
CSA processing results of the outer race fault signal: (a) reconstructed signal; (b) envelope spectrum.

In Fig 15(a), strong noise leads to significant dispersion of the impulsive trajectories in the TFR obtained using TMSST, thereby weakening the concentration of TF energy. As shown in Fig 15(b), the signal reconstructed by TMSST lacks obvious periodic impulsive structures, while part of the fault-related transient components is obscured by noise interference. Consequently, Fig 15(c) shows that the characteristic frequency corresponding to the outer race fault cannot be accurately identified from the results.

**Fig 15:**
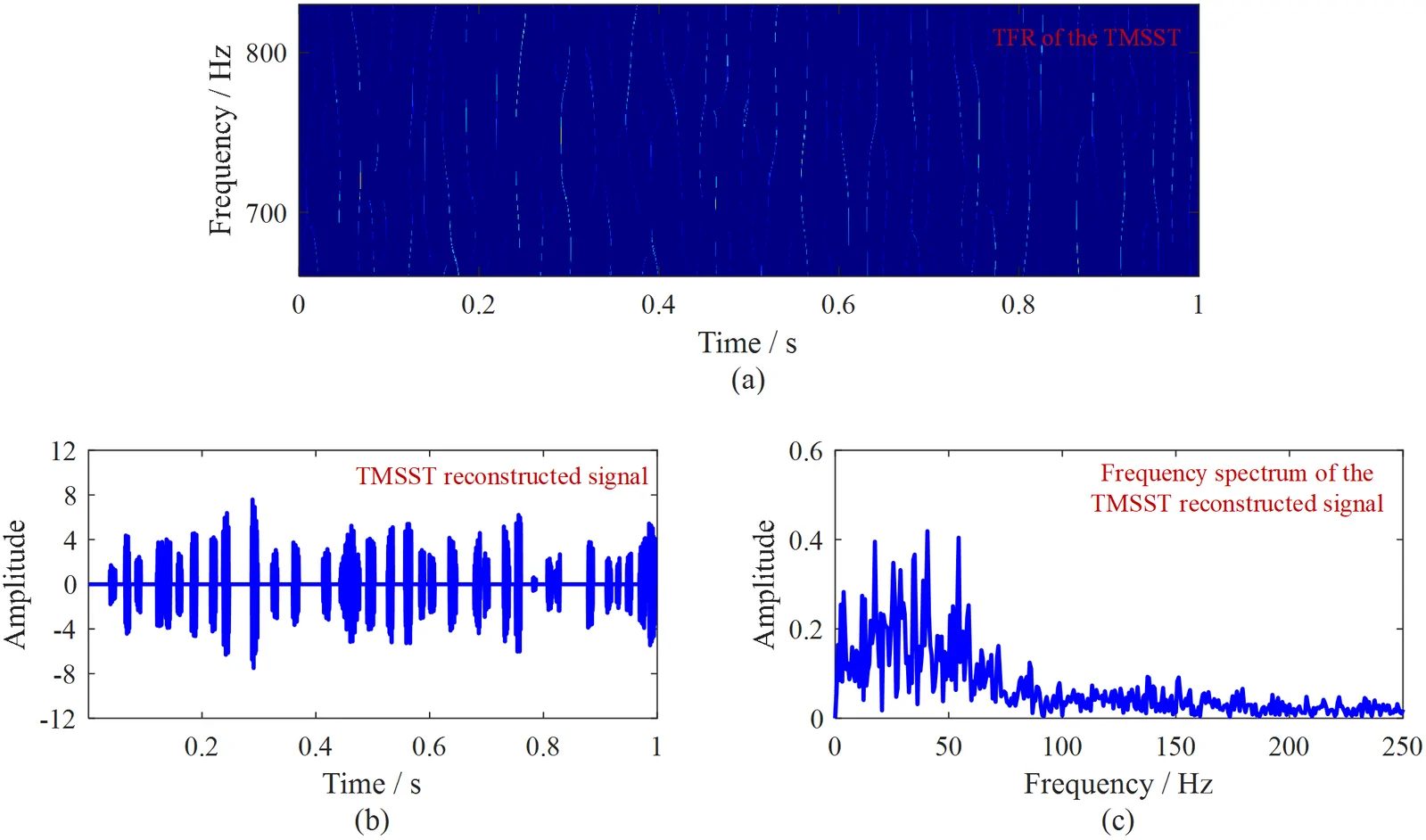
TMSST results for the outer race fault signal: (a) TFR obtained by TMSST; (b) extracted fault component; (c) envelope spectrum.

Fig 16 presents the L1-TMSST analysis results for the experimental outer race fault signal. In Fig 16(a), the TF representation obtained by L1-TMSST reveals a series of periodically distributed fault-related transient trajectories. However, the energy concentration of these trajectories remains relatively weak, and local attenuation can still be observed. The reconstructed signal in Fig 16(b) exhibits a distinct periodic impulsive pattern, confirming that L1-TMSST is capable of extracting the outer race fault-related transient components. Nevertheless, the amplitudes of the reconstructed impulses are relatively small, indicating that part of the transient energy is attenuated during the sparse reconstruction process of L1-TMSST.

**Fig 16:**
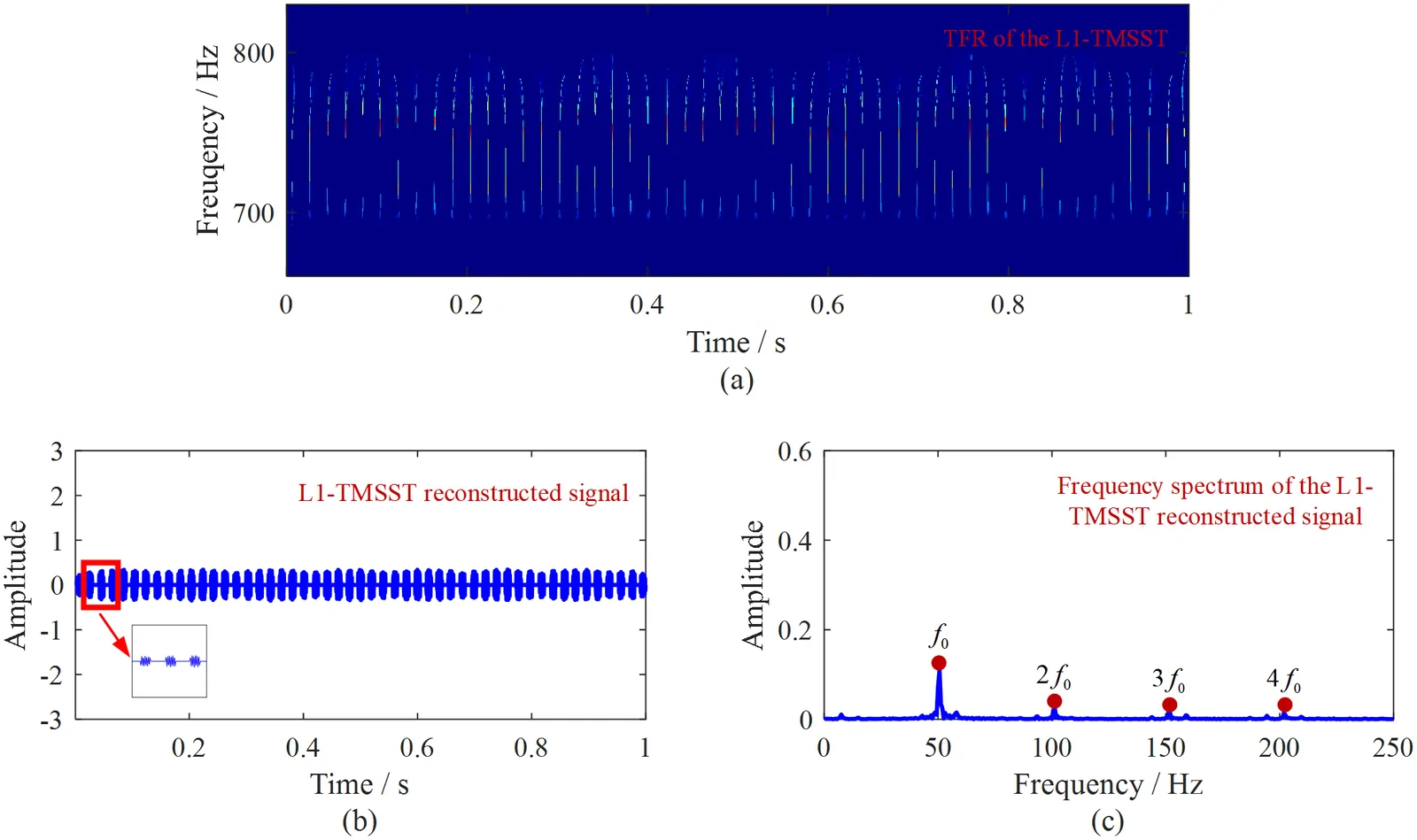
L1-TMSST results for the outer race fault signal: (a) TFR obtained by L1-TMSST; (b) extracted fault component; (c) envelope spectrum.

Fig 16(c) clearly reveals the outer race fault characteristic frequency $f_{\text{0}}$ and its harmonics. However, compared with the CSA-TMSST results in Fig 17, the fault-related TF trajectories and reconstructed impulses obtained by L1-TMSST exhibit lower energy intensity, and the amplitudes of the corresponding spectral peaks are also less pronounced. These observations suggest that L1-TMSST may introduce excessive shrinkage of weak transient coefficients while suppressing noise, thereby impairing the preservation of fault-related impulsive energy.

**Fig 17:**
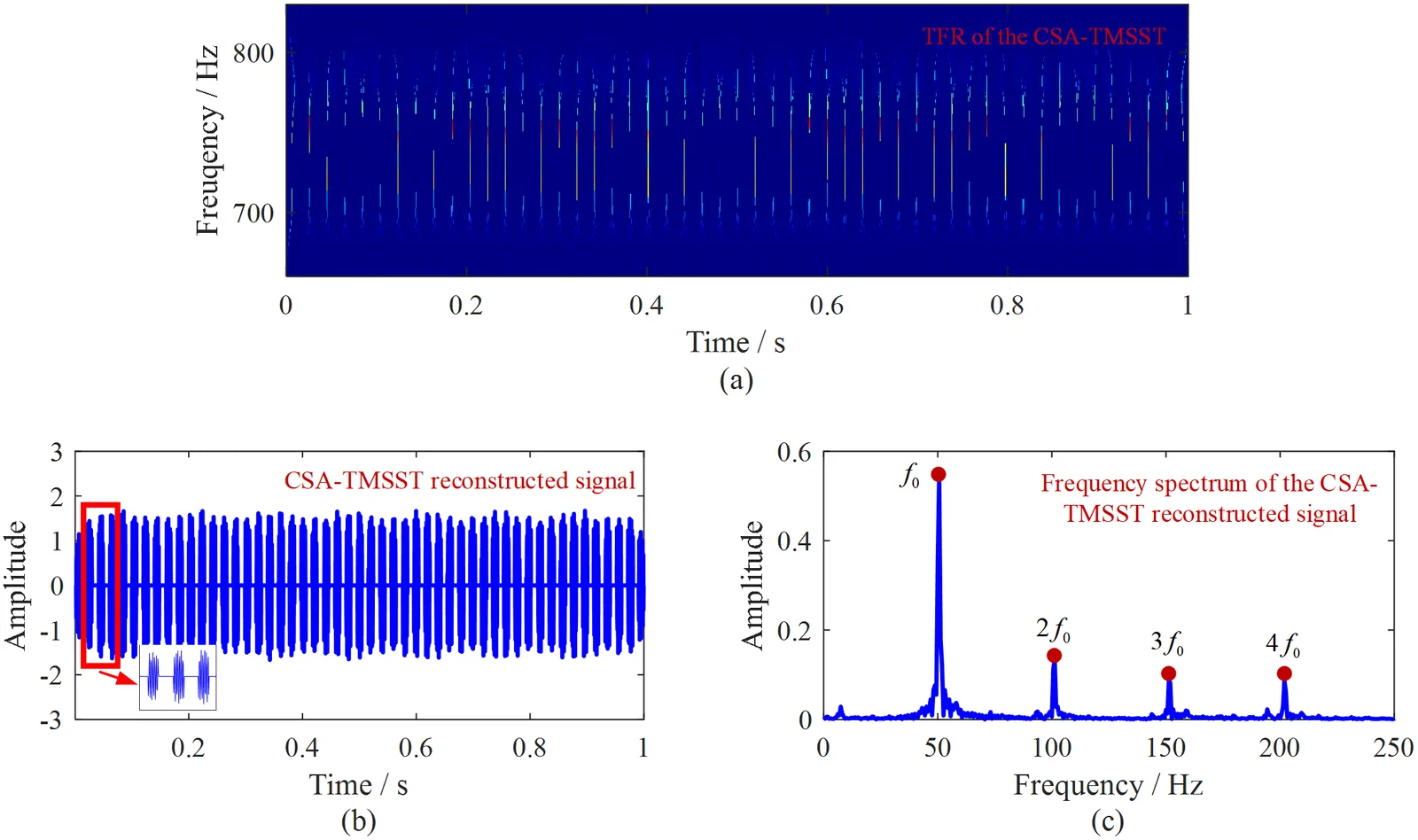
CSA-TMSST results for the outer race fault signal: (a) TFR obtained by CSA-TMSST; (b) extracted fault component; (c) envelope spectrum.

In contrast, Fig 17(a) shows that CSA-TMSST produces more continuous and pronounced fault-related impulsive trajectories with a clearer periodic distribution. The reconstructed signal in Fig 17(b) contains clear and regularly repeated fault impulses with substantially enhanced amplitudes, demonstrating improved preservation of the fault-related transient components. Moreover, Fig 17(c) clearly identifies the characteristic frequency $f_{\text{0}}\approx 50.6\text{Hz}$ and its harmonics, with the fundamental component being substantially more prominent than that obtained by L1-TMSST. The identified characteristic frequency agrees well with the theoretical outer race fault frequency, confirming the effectiveness of CSA-TMSST for weak bearing fault diagnosis under strong noise conditions.

### 4.2 Verification with measured wind turbine vibration data

The bearing vibration dataset analyzed in this section was obtained from a 1.5 MW direct-drive wind turbine. The system mainly consists of the main shaft, the rotating shaft, a generator stator, and a generator rotor and so on. Among them, the main shaft bearing consists of the front and rear bearings with the models of BT2-8168 and NJ28/770, respectively.

In this study, the horizontal vibration response of the rear bearing on the main shaft is investigated. Table 2 summarizes the mechanical frequency parameters of the wind turbine system. During the experiment, the rotor is operated at a constant rotational speed of 17.3 rpm, while vibration data are sampled at 1280 Hz.

**Table 2:** Wind turbine mechanical characteristic frequencies.

Parts	Mechanical characteristics	Feature frequency (Hz)
Main bearing (BT2–8168)	Inner race defect frequency Outer race defect frequency Ball defect frequency	6.02 5.22 3.59
Rear bearing (NJ28/770)	Inner race defect frequency Outer race defect frequency Ball defect frequency	7.42 6.71 5.69

Fig 18 shows the waveform of the original vibration signal and its corresponding envelope spectrum. As shown in Fig 18(a) and (b), strong background noise severely interferes with the signal, making the fault characteristic frequencies difficult to identify. Subsequently, vibration signals from the wind turbine bearing are processed and compared using EMD, VMD, CSA, TMSST, and L1-TMSST. Fig 19 illustrates the decomposition characteristics of the wind turbine vibration signal obtained through EMD.

**Fig 18:**
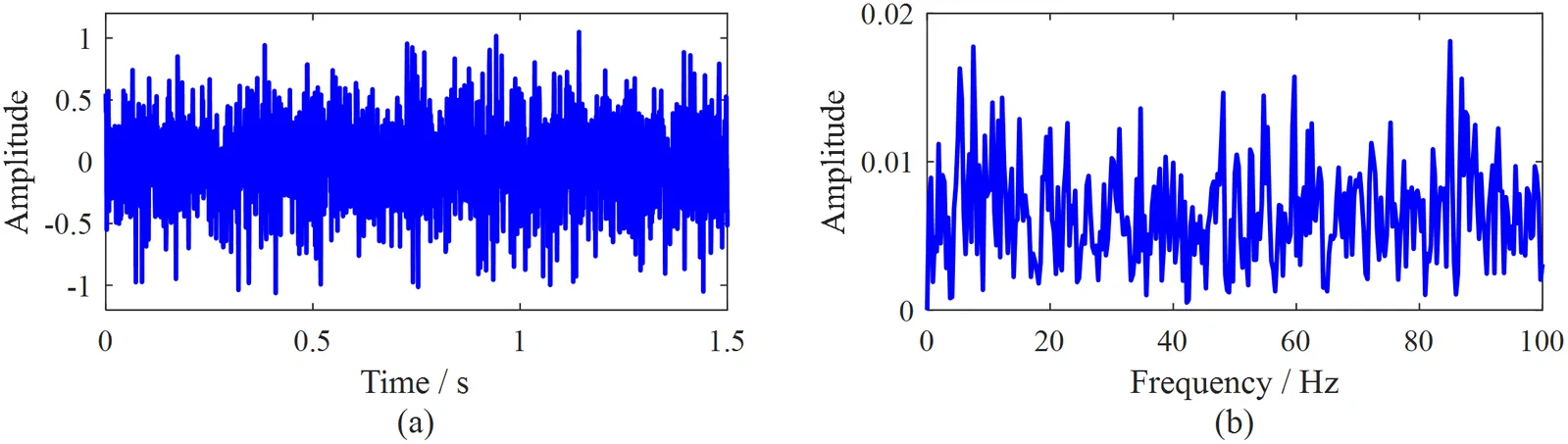
Vibration signal of the wind turbine bearing: (a) waveform; (b) envelope spectrum.

**Fig 19:**
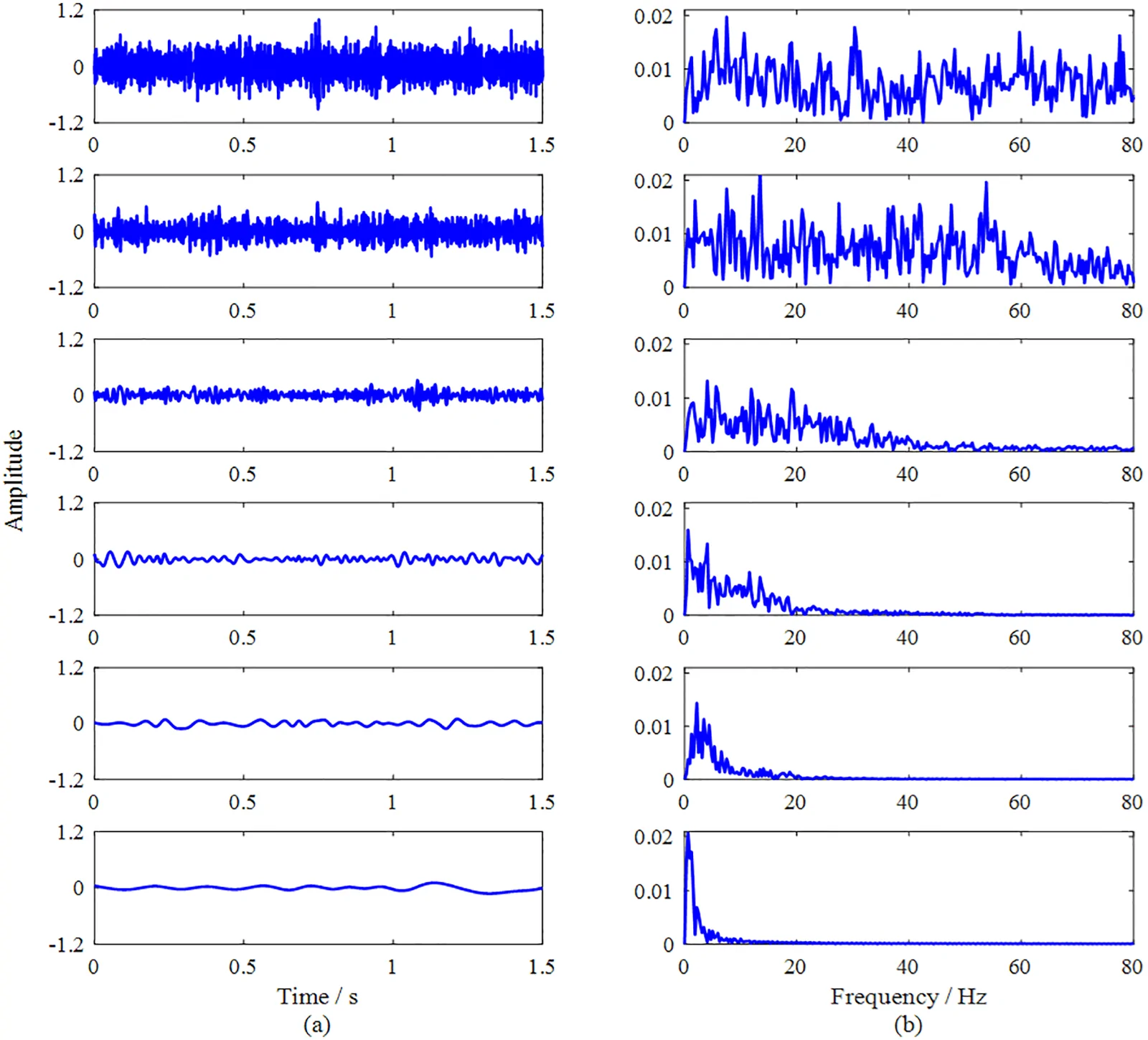
EMD results for the wind turbine bearing fault signal: (a) IMFs; (b) envelope spectra of the IMFs.

In Fig 19(a), the six IMF components indicate that EMD cannot clearly characterize the weak impulsive components caused by early faults. Furthermore, in Fig 19(b), the envelope spectrum results of the six IMF components fail to reveal the fault characteristic frequency, reflecting the limited performance of EMD for weak fault feature extraction in noisy environments. Similarly, as shown in the VMD results in Fig 20(a) and (b), the six modes obtained by VMD fail to effectively capture the transient impulsive features associated with the bearing defect. In addition, the envelope spectra of the extracted modes fail to clearly reveal the fault characteristic frequency.

**Fig 20:**
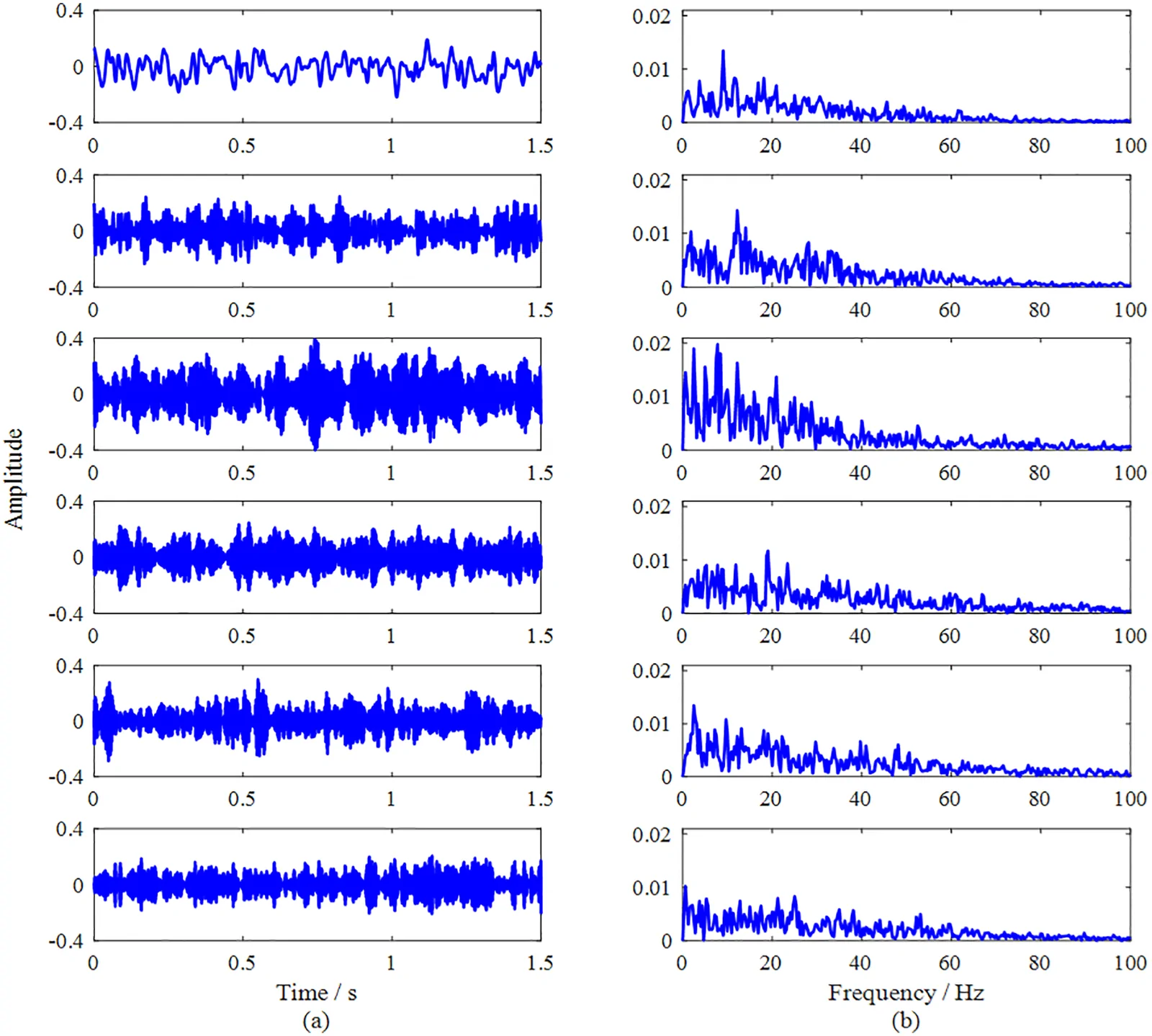
VMD results for the wind turbine bearing fault signal: (a) decomposed modes; (b) corresponding envelope spectra.

Fig 21(a) shows that, although CSA suppresses part of the broadband background noise, considerable residual interference remains in the reconstructed signal, making the weak periodic impulsive components difficult to distinguish clearly. As shown in Fig 21(b), the expected fault characteristic frequency can be identified, indicating that CSA can preserve and enhance fault-related transient information to a certain extent. However, the fault characteristic frequency is still not sufficiently prominent in the spectrum, and its corresponding harmonic structure remains incomplete.

**Fig 21:**
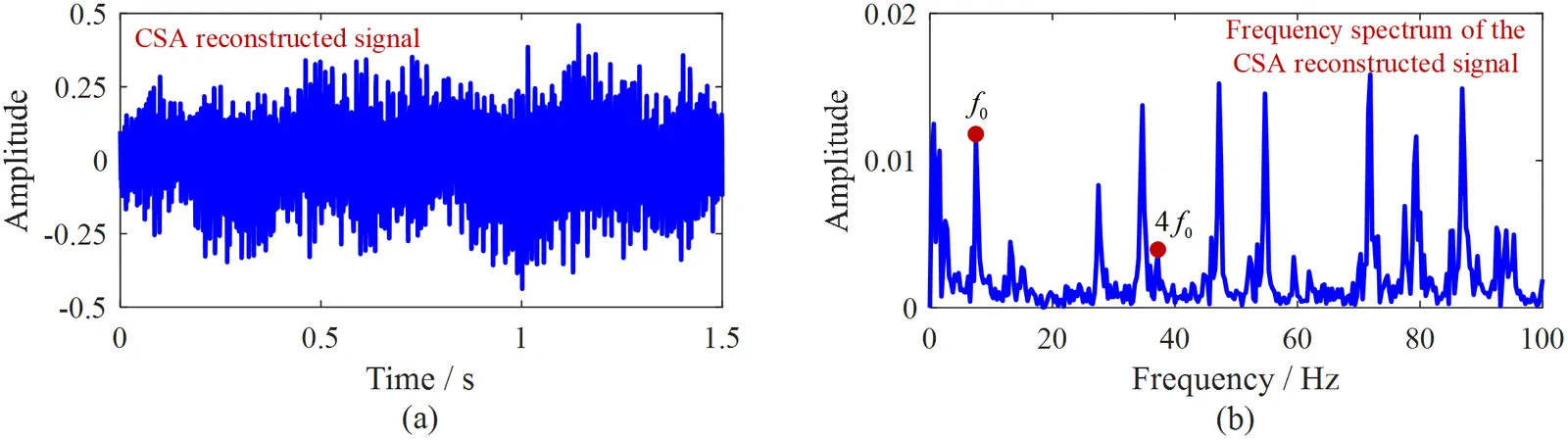
CSA results for the wind turbine bearing fault signal: (a) reconstructed signal; (b) corresponding envelope spectrum.

In addition, several non-fault-related spectral peaks with amplitudes comparable to or even higher than those of the fault-related peaks are still present, thereby complicating reliable fault feature identification. These results indicate that CSA can provide preliminary enhancement of weak fault-related transient components. However, CSA alone remains insufficient to effectively separate and highlight the periodic fault-related components in wind turbine bearing signals under complex background interference.

The TFR obtained by TMSST is further presented in Fig 22. In Fig 22(a), strong noise substantially degrades the TF energy concentration of TMSST, resulting in discontinuous and dispersed trajectories associated with the weak fault impulses. Fig 22(b) shows that it is still difficult to observe clear periodic impulsive structures in the impulsive signal reconstructed by TMSST. In addition, the characteristic frequency associated with the bearing fault is not clearly distinguishable in Fig 22(c). These results indicate that TMSST has difficulty in effectively extracting and diagnosing early weak bearing fault features.

**Fig 22:**
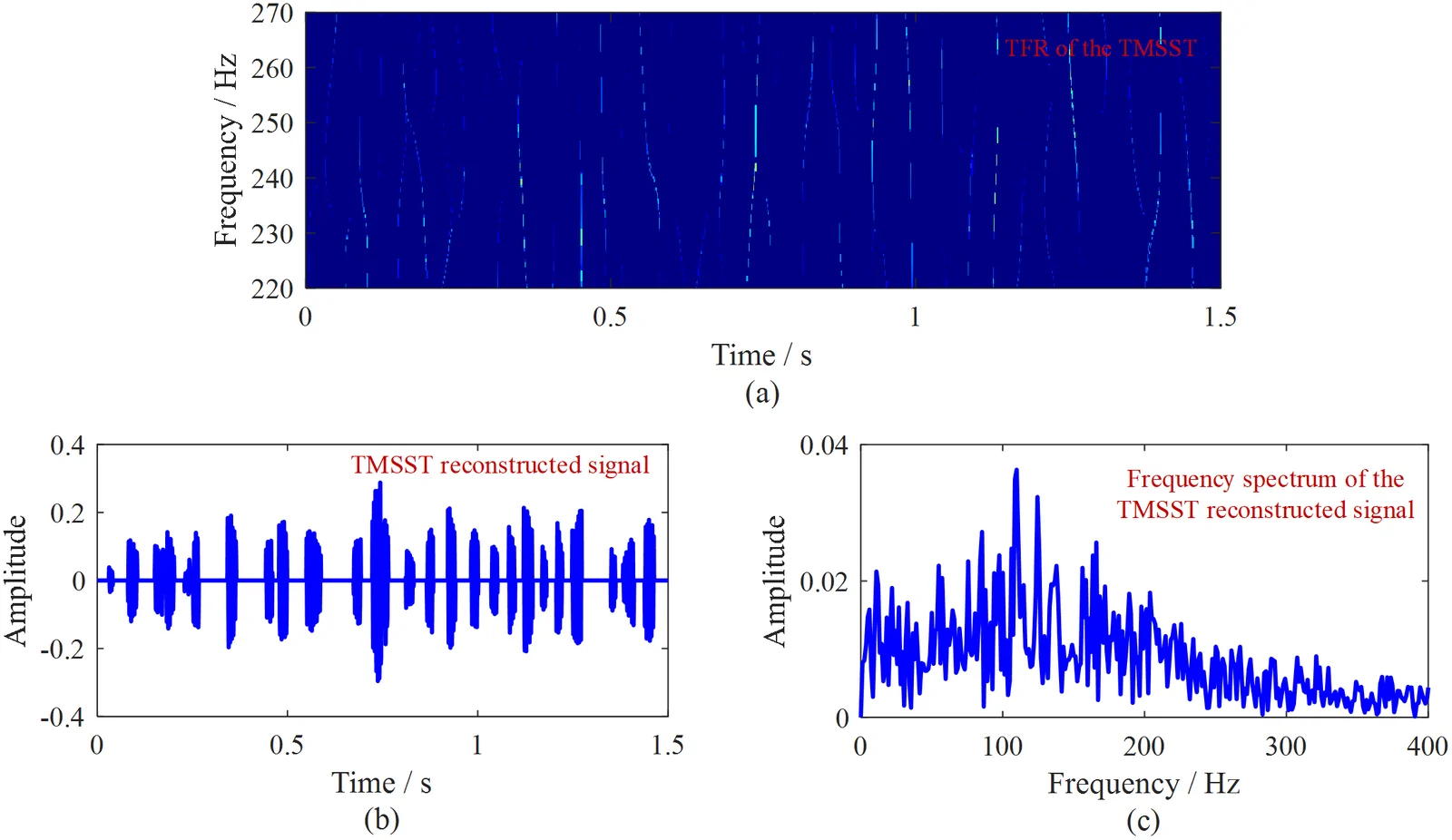
TMSST results for the wind turbine vibration signal: (a) TFR obtained via TMSST; (b) extracted impulsive component; (c) corresponding envelope spectrum.

Fig 23 presents the analysis results obtained using L1-TMSST. In Fig 23(a), several periodically distributed fault-related transient trajectories can be observed in the TF plane. However, these trajectories still exhibit noticeable energy attenuation and local discontinuities, and the TF energy associated with some impacts remains relatively weak. Fig 23(b) shows that the reconstructed signal generally exhibits a periodic impulsive pattern. Nevertheless, a distinct temporal misalignment and waveform distortion can be observed near 1.1 s, indicating that the ability of L1-TMSST to accurately reconstruct some weak transient components remains limited under strong noise conditions. Furthermore, the fault characteristic frequency $f_{\text{0}}$ and its harmonic components can be identified in Fig 23(c), demonstrating that L1-TMSST retains some capability for fault feature extraction. However, the amplitudes of the fault-related spectral peaks are generally low, and the local misalignment observed in the reconstructed impulses further indicates that, although L1-TMSST can achieve fault feature identification, its ability to fully preserve and accurately reconstruct weak transient components remains limited.

**Fig 23:**
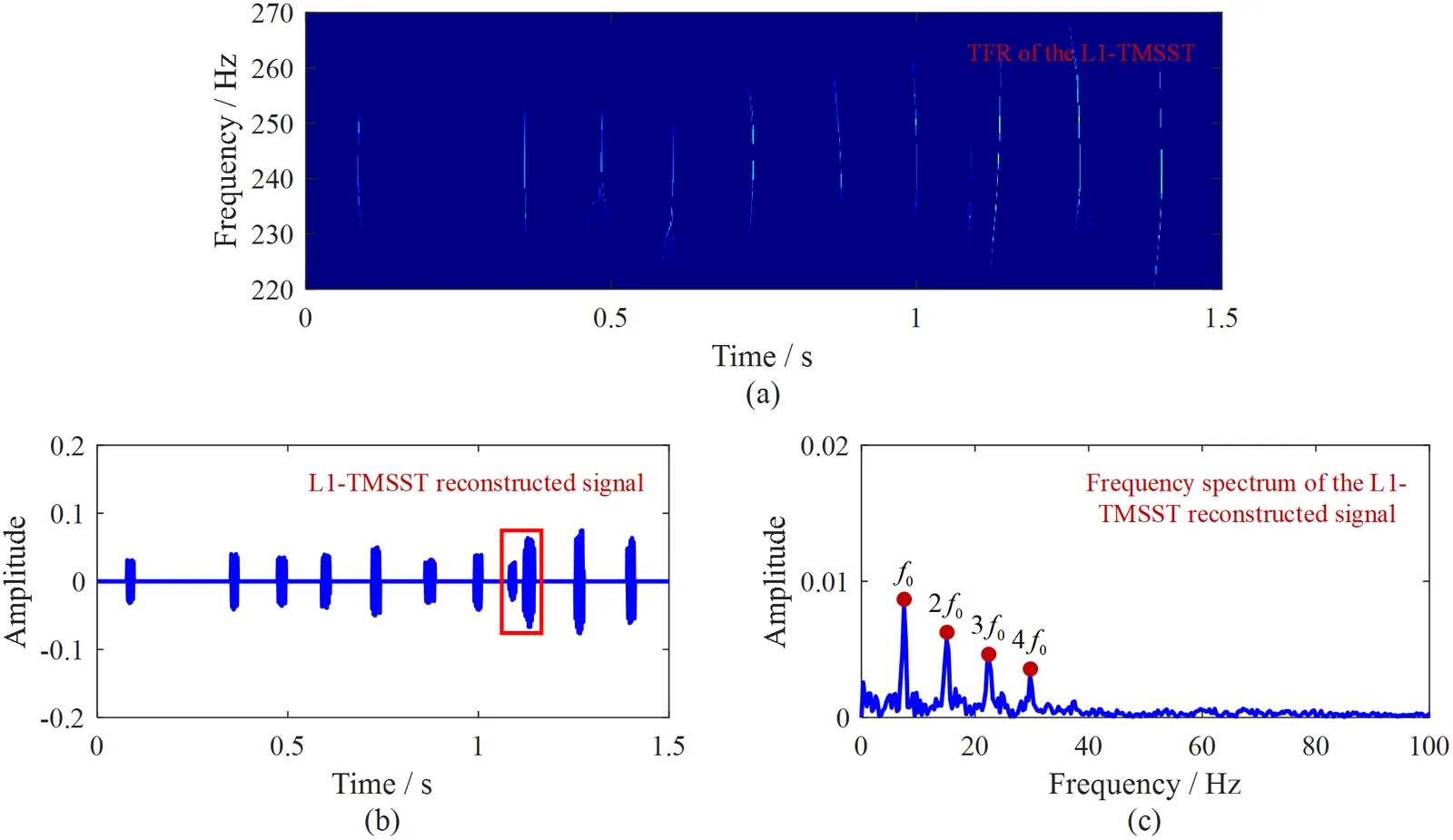
L1-TMSST results for the wind turbine vibration signal: (a) TFR obtained via L1-TMSST; (b) extracted impulsive component; (c) corresponding envelope spectrum.

In contrast, the CSA-TMSST results shown in Fig 24(a) exhibit more complete and regularly distributed fault-related transient trajectories in the TF plane. The reconstructed signal in Fig 24(b) contains a series of clearly separated periodic impulses, with an average time interval of $T_{\text{0}}\approx 0.135\text{s}$. Accordingly, the corresponding fault characteristic frequency is estimated as $f_{\text{0}}\approx 7.41\text{Hz}$. Furthermore, the envelope spectrum in Fig 24(c) exhibits a prominent fundamental frequency component at approximately 7.49 Hz, together with several clearly identifiable harmonics. Both estimates are in close agreement with the theoretical rear-bearing inner-race fault characteristic frequency of 7.42 Hz listed in Table 2. Compared with L1-TMSST, CSA-TMSST produces more complete and continuous transient trajectories, better preserves the amplitudes of fault-related impulses, and yields more prominent fault-related spectral peaks, thereby demonstrating superior capability for weak fault feature enhancement and extraction. From the perspective of practical fault diagnosis, computational efficiency is an important consideration for iterative signal-processing methods. Therefore, the execution times of EMD, VMD, CSA, TMSST, L1-TMSST, and CSA-TMSST were compared using the measured wind turbine bearing signal. All methods were implemented in MATLAB R2024b and executed on the same computer equipped with a 12th Gen Intel Core i5-12500H processor (2.50 GHz) and 16 GB of RAM. The corresponding computational times are summarized in Table 3.

**Table 3:** Computational times of the compared methods for the wind turbine bearing signal.

Method	EMD	VMD	CSA	TMSST	L1-TMSST	CSA-TMSST
Computational time (s)	0.6898	1.0435	0.9528	7.7045	2.5318	3.6912

**Fig 24:**
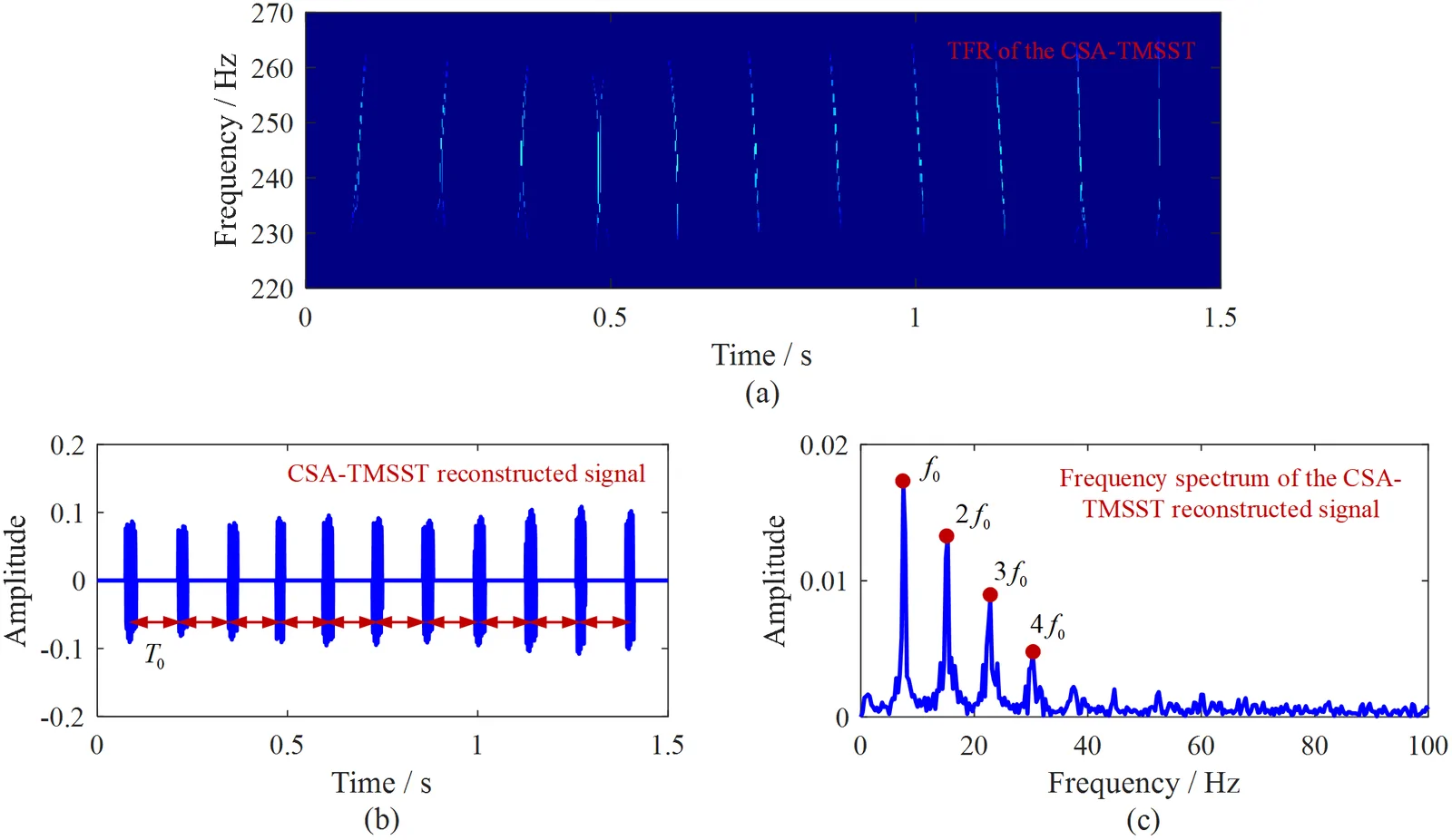
CSA-TMSST results for the wind turbine vibration signal: (a) TFR obtained via CSA-TMSST; (b) extracted impulsive component; (c) corresponding envelope spectrum.

As shown in Table 3, EMD, CSA, and VMD require relatively short computational times of 0.6898 s, 0.9528 s, and 1.0435 s, respectively, reflecting their lower computational burden under the present implementation. However, as demonstrated by the preceding results, these methods exhibit limited capability in extracting weak fault-related transient components under strong background interference. L1-TMSST requires 2.5318 s, whereas CSA-TMSST requires 3.6912 s because of the additional GMC-based non-convex sparse reconstruction procedure. Nevertheless, CSA-TMSST remains considerably more computationally efficient than direct TMSST, whose computational time reaches 7.7045 s. Moreover, the additional computational cost is accompanied by improved preservation of weak transient impulses, higher TF energy concentration, and clearer fault characteristic frequencies. Therefore, in practical industrial deployment, computational efficiency should be evaluated jointly with diagnostic accuracy, considering the trade-off between computational burden and fault-feature extraction performance [31,32].

## 5 Conclusions

To enhance weak fault feature extraction in wind turbine bearings under strong noise interference, a novel CSA-TMSST is proposed. The method integrates non-convex sparse optimization with TMSST to enhance transient fault impulses and improve TF energy concentration. The incorporation of a GMC-based sparse regularization model enables the extraction and reconstruction of fault-related impulsive signatures from raw vibration signals, resulting in improved signal sparsity and enhanced feature characterization. Both numerical simulations and experimental investigations indicate that CSA-TMSST can effectively suppress strong background noise while preserving weak transient fault information. In comparison with existing TFA techniques, the approach exhibits superior performance in TF energy concentration, fault feature enhancement, and early weak fault identification capability.
